# Substrate recognition and catalysis by LytB, a pneumococcal peptidoglycan hydrolase involved in virulence

**DOI:** 10.1038/srep16198

**Published:** 2015-11-05

**Authors:** Palma Rico-Lastres, Roberto Díez-Martínez, Manuel Iglesias-Bexiga, Noemí Bustamante, Christine Aldridge, Dusan Hesek, Mijoon Lee, Shahriar Mobashery, Joe Gray, Waldemar Vollmer, Pedro García, Margarita Menéndez

**Affiliations:** 1 Instituto de Química-Física Rocasolano, Consejo Superior de Investigaciones Científicas, Serrano 119, 28006, Madrid, Spain; 2 CIBER de Enfermedades Respiratorias (CIBERES), Instituto de Salud Carlos III (ISCIII), Madrid, Spain; 3 Centro de Investigaciones Biológicas, Consejo Superior de Investigaciones Científicas, Ramiro de Maeztu 9, 28040, Madrid, Spain; 4 Centre for Bacterial Cell Biology, Institute for Cell and Molecular Biosciences, Newcastle University, Newcastle upon Tyne NE2 4AX, United Kingdom; 5 Department of Chemistry and Biochemistry, University Notre Dame, Notre Dame, Indiana 46556, United States; 6 Institute for Cell and Molecular Biosciences, Newcastle University, Newcastle upon Tyne NE2 4AX, United Kingdom

## Abstract

*Streptococcus pneumoniae* is a major cause of life-threatening diseases worldwide. Here we provide an in-depth functional characterization of LytB, the peptidoglycan hydrolase responsible for physical separation of daughter cells. Identified herein as an *N*-acetylglucosaminidase, LytB is involved also in colonization and invasion of the nasopharynx, biofilm formation and evasion of host immunity as previously demonstrated. We have shown that LytB cleaves the GlcNAc-*β-*(1,4)-MurNAc glycosidic bond of peptidoglycan building units. The hydrolysis occurs at sites with fully acetylated GlcNAc moieties, with preference for uncross-linked muropeptides. The necessity of GlcN acetylation and the presence of a single acidic moiety (Glu585) essential for catalysis strongly suggest a substrate-assisted mechanism with anchimeric assistance of the acetamido group of GlcNAc moieties. Additionally, modelling of the catalytic region bound to a hexasaccharide tripentapeptide provided insights into substrate-binding subsites and peptidoglycan recognition. Besides, cell-wall digestion products and solubilisation rates might indicate a tight control of LytB activity to prevent unrestrained breakdown of the cell wall. Choline-independent localization at the poles of the cell, mediated by the choline-binding domain, peptidoglycan modification, and choline-mediated (lipo)teichoic-acid attachment contribute to the high selectivity of LytB. Moreover, so far unknown chitin hydrolase and glycosyltransferase activities were detected using GlcNAc oligomers as substrate.

*Streptococcus pneumoniae*, the pneumococcus, is a Gram-positive aerotolerant bacterium that colonizes the nasopharyngeal cavity of children and adults. It is the main cause of several community-acquired infections, including bacteraemia, meningitis, pneumonia, and otitis media^1^,^2^. This bacterium causes 1–2 million deaths per year, most of which occur in developing countries^3^. Sickle-cell anaemia, HIV infection, and a variety of chronic organ failure conditions increase the risk of serious pneumococcal disease^4^. The peptidoglycan (PG), a major component of the bacterial cell wall, is essential for shape maintenance and protects bacteria from bursting by the turgor^5^. Typical PG consists of a polymeric network comprised of chains of *N*-acetylglucosamine-*β*-(1,4)-*N*-acetylmuramic acid (GlcNAc-MurNAc) connected via short peptides bound to the lactyl carboxyl group of MurNAc. In *S. pneumoniae* a variable proportion of *N*-acetylglucosamine residues become deacetylated to glucosamine, contributing to the bacterial resistance to the action of the host lysozyme, an important element of the innate immunity^6^,^7^. The pneumococcal cell wall also contains unusually complex (lipo- and wall-) teichoic acids (TAs) with covalently linked phosphocholine residues^8^. The PG is continuously remodelled throughout the cell cycle and its complex fine structure illustrates the PG modification and maturation during cell growth^7^,^9^. Its growth requires the combined action of biosynthetic and hydrolytic enzymes, which makes them appropriate targets for the design of novel antibacterials. Pneumococcal cells divide in parallel planes perpendicular to their long axis^10^ and peripheral PG biosynthesis extends from the middle of dividing cells to the equator of what will become the daughter cell, whereas septal biosynthesis creates a PG cross-wall between the daughter cells^11^,^12^.

LytB is a pneumococcal non-autolytic PG hydrolase identified in our work as a glucosaminidase that cleaves the GlcNAc-*β*(1,4)-MurNAc glycosidic bond of the PG backbone. LytB is responsible for the physical separation of daughter cells, the final event of cell-division cycle^13^. Furthermore, it is a virulence factor involved in the adhesion to and invasion of respiratory epithelial cells^14^,^15^,^16^,^17^, evasion of phagocytosis by alveolar macrophages^15^, and knock-out of the *lytB* gene decreases by ~45% the ability of R6 cells to form biofilms^18^. Hence, LytB is considered a putative vaccine/drug target. As shown in Fig. 1a, LytB exhibits a modular structure that comprises an N-terminal choline-binding domain (LytB_CBD_) and a C-terminal domain (LytB_GH73_) belonging to the GH73 family of glycoside hydrolases (Pfam entry PF01832). They are connected through a WW-like domain (LytB_WW_) and an SH3b domain (LytB_SH3b_), both relevant for substrate recognition and LytB-mediated host-cell adhesion^17^. The crystal structure of a LytB fragment (LytB_CAT_) comprising all but the N-terminal LytB_CBD_ domain has been recently reported^17^. Like the three other members of the GH73 family with known structures, LytB_GH73_ comprises an α-helical domain structurally related to those of glycoside hydrolases from families GH19, GH22, GH23 and GH103^17^,^19^,^20^,^21^. The LytB_CBD_ domain, essential for PG hydrolysis, recognizes the choline moieties of TAs associated to the pneumococcal envelope^13^ and is composed of tandemly arranged choline-binding repeats (CBRs) (Pfam accession code PF01473) predicted to form a left-handed *β*-solenoid, where choline would bind at the interface of two consecutive CBRs^22^,^23^,^24^. The number of LytB CBRs depends on the particular strain, ranging from 18 (in the non-encapsulated R6 strain) to 12 (in some atypical strains)^25^. The physiological function of LytB as a PG hydrolase was confirmed by its chain-dispersing activity on long chains of LytB-deficient cultures^13^,^17^, though the average chain-length of *lytB* mutant cells might also depend on the serotype and growth conditions^13^,^16^,^17^,^26^. Interestingly, LytB exhibits a rather low specific activity on purified pneumococcal cell walls when compared to LytC or LytA (the pneumococcal autolysins) and cleaves a limited number of glycosidic bonds^13^. This partial hydrolysis of purified cell walls and its non-autolytic character might indicate a restricted recognition and/or degradation of the PG substrate. The structural determinants accounting for this behavior remain however unknown.

In the present work we have investigated the LytB-mediated hydrolysis, including substrate recognition and specificity, catalytic reaction and cellular localization by using different approaches, *i.e.*, analysis of cell-wall degradation products from different pneumococcal strains, *in vitro* degradation of small PG analogues, molecular modelling, and correlation of serial deletions of the LytB_CBD_ domain with cellular localization and dispersing activity. Our results reveal that LytB cleaves the GlcNAc-*β*(1,4)-MurNAc glycosidic bonds and that hydrolysis takes place at PG sites with fully acetylated GlcNAc moieties, with preference for the uncross-linked muropeptides. The inability of LytB to hydrolyse deacetylated muropeptides and the existence of only one single acidic residue (Glu585) essential for catalysis strongly suggests that the reaction takes places via a substrate-assisted mechanism, further supporting the notion that GH73 family enzymes do not share a unique catalytic mechanism^21^. Furthermore, modelling of LytB_CAT_ bound to a hexasaccharide tripentapeptide (GMPP)_3_ provided structural insights into the substrate-binding subsites and into catalysis. We also show that LytB choline-independent localization at the cell poles requires the presence of the LytB_CBD_ domain. Together, our data help to understand how the architectural complexity of PG hydrolases contributes to finely tune substrate specificity.

## Results

### Identification of LytB catalytic residues

**Figure 1:**
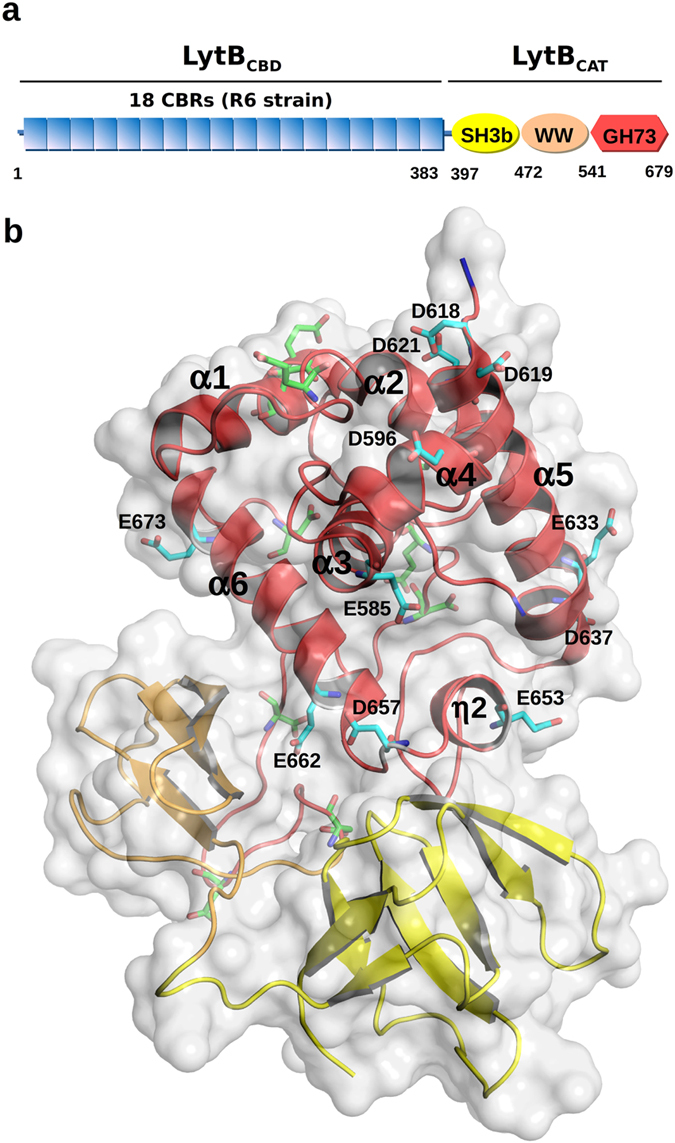
Molecular architecture of the LytB glucosaminidase from *S. pneumoniae* R6. (**a**) Scheme of LytB domain composition. Red hexagon represents the GH73 domain (LytB_GH73_), yellow and salmon ellipses the SH3b (LytB_SH3b_) and WW (LytB_WW_) domains, respectively, which conform LytB_CAT_. Blue rectangles represent the CBRs of the CBD domain (LytB_CBD_). Numbers indicate the beginning and the end of each domain. (**b**) Cartoon representation of LytB_CAT_ comprising LytB_GH73_, LytB_WW_ and LytB_SH3b_ domains coloured in red, salmon and yellow, respectively. Acidic residues mutated to alanine are shown in stick representation except for Asp607 which is located in the flexible region of LytB_GH73_ and does not appear the X-ray structure (carbon atoms in blue, oxygen in red and nitrogen in blue). Non substituted acidic residues of LytB_CAT_ located out of the substrate binding groove are depicted in with carbon atoms in green.

The LytB_GH73_ catalytic domain comprises six *α*-helices (*α*-1 to *α*-6), a 3_10_ helix (*η*-2), and a flexible segment of 11-residues connecting helices *α*-4 and *α*-5 (from Ala605 to Lys615 in the R6 sequence numbering), which was missing in the electron-density maps^17^ (Fig. 1b). Though shorter, this flexible segment, together with helix *α*-4, structurally corresponds to the long *β*-hairpin and loops forming the *β*-lobe that helps to shape the catalytic groove in Auto and FlgJ GH73 glucosaminidases produced by *Lysteria monocytogenes* and *Sphingomonas sp*. strain A1, respectively^19^,^20^. Members of GH73 family share with structurally related glycoside hydrolases a conserved glutamate near the C-terminus of helix *α*-3 (Glu585 in LytB), proposed as the catalytic proton-donor (see Supplementary Fig. S1). In contrast, identification of a second carboxylate which could act as the nucleophile that stabilises the oxocarbenium intermediate or forms a covalent intermediate in retaining glycosidases, or the general base that activates the water molecule that would attack the oxocarbenium intermediate in the inverting enzymes, has been inconclusive with varying results from different proteins and families^19^,^20^,^21^,^27^,^28^,^29^,^30^,^31^. Moreover, the coexistence of inverting and substrate-assisted mechanisms in glycoside hydrolases of GH73 family has been suggested recently^21^. This latter mechanism does not require a second catalytic carboxylate as the reaction intermediate is stabilized by the *N*-acetyl group of the sugar moiety at position −1 (bond cleavage occurs between positions −1 and +1) and can account for retention of the anomeric configuration during reaction in glycoside hydrolases without a second catalytic carboxylate^32^.

**Figure 2:**
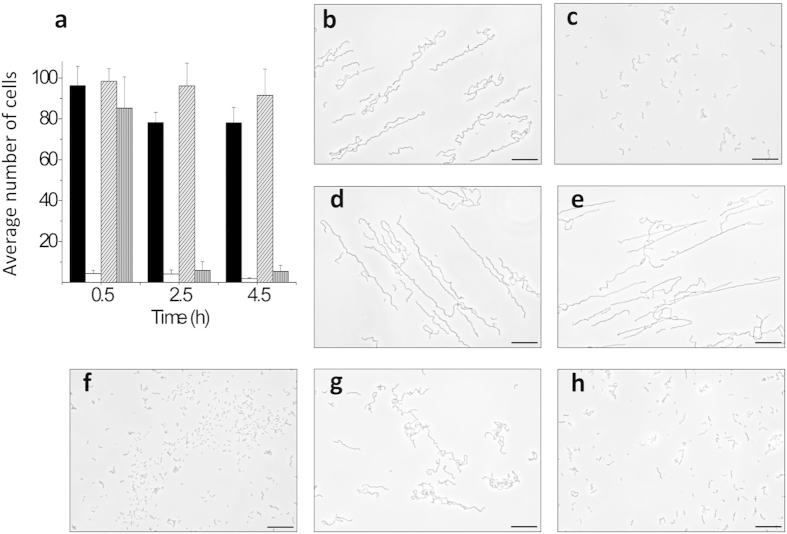
Chain dispersing activity of the wild type and alanine mutants of LytB. R6B cultures (OD_550_ ~ 0.2) were treated at 37 °C with the purified proteins (10 μg/ml) and aliquots were removed, at different times, to examine the culture morphology and determine the average chain length by using phase-contrast microscopy. (**a**) Chain dispersion by LytB wild type (white bars), E585A (hatched bars) and D657A (vertical-stripped dark-grey bars) LytB mutants. Untreated cells (black bars), used as controls, were also examined. Error bars represent standard deviations. (**b**) Morphology of untreated cells. (**c**,**d**,**f**,**g**) Morphology of cells after 30 min of incubation with LytB wild type, D585A, D619A or D657A mutants, respectively. (**e**,**h**) Morphology of cells after 4.5 h of incubation with D585A or D657A mutants, respectively. Bars, 25 μm.

To search for the acidic residue potentially acting either as a nucleophile or a general base during catalysis, all aspartic and glutamic acids of LytB_GH73_ located in the substrate-binding groove or within its vicinity (Glu585, Asp596, Asp607, Asp618, Asp619, Asp621, Glu633, Asp637, Glu653, Asp657, Glu662 and Glu673) were individually replaced by alanine using site-directed mutagenesis (“alanine scanning”). The unchanged acidic residues (Glu558, Glu566, Glu568, Glu569 and Asp679) are more than 21 Å away or at the opposite face of the catalytic cleft (Fig. 1b). The purified mutant proteins were examined for cell-dispersing activity using cultures of R6B, a LytB-deficient strain that forms long chains of cells (Fig. 2a,b). Only diplococci and very short chains were observed after treating R6B cultures for 30 min with the wild-type LytB (Fig. 2a–c), whereas the cell-chain length remained invariant after 5 h of incubation with the E585A mutant (Fig. 2d,e), in agreement with its essential role in catalysis in the wild-type enzyme^17^. All the other mutants, except D657A, behaved like the wild-type protein, as illustrated for the D619A variant in Fig. 2f, excluding their participation in catalysis. In contrast, addition of the D657A mutant resulted in a minor dispersion of daughter cells during the first 30 min of incubation, although diplococci and very short chains became predominant after a 4.5-h treatment (Fig. 2g,h), revealing a significant reduction of the chain dispersion rate. The CD spectra of E585A and D657A mutants superimposed with that of the wild-type protein, dispelling the possibility that the activity loss was due to the mutant proteins fold differently than the wild-type protein (Supplementary Fig. S2). The Asp657 residue belongs to a highly conserved motif (YATD) in GH73 family (Supplementary Fig. S1), making it a good candidate for the aforementioned nucleophile/base. However, its distance (7.9 Å) to the carboxyl group of the Glu585, the proton donor, is between those reported for retaining (4–5 Å) or inverting (9–11 Å) enzymes^33^.

Furthermore, substitution of the equivalent residue (D1335) in the glucosaminidase domain of Atl_WM_ of *Staphylococcus warneri* by alanine or asparagine yielded nearly fully active mutant proteins^27^. Altogether, these results further indicated the absence of conservation of the nucleophile/base catalyst in the GH73 family or, alternatively, the existence of a substrate-assisted mechanism, as has been proposed for the GH23, GH103 and GH104 families^31^.

## Substrate Specificity of LytB

### Reaction products of *in vitro* cell-wall degradation

**Figure 3:**
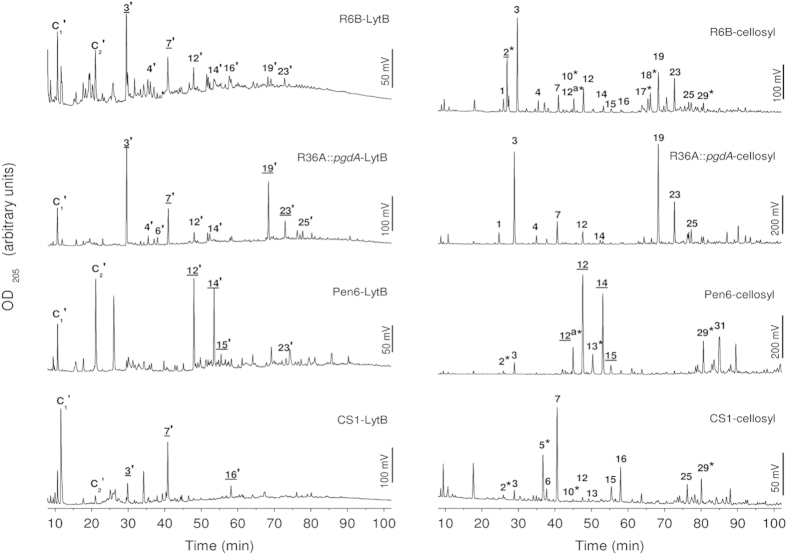
Comparison of the pattern of PG fragments released with LytB or cellosyl from cell wall or PG from different pneumococcal strains. Reduced PG fragments solubilized by PG-digestion with cellosyl or cell-wall digestion with LytB were separated on a Prontosil C18 column, and the OD_205_ of the eluate was monitored. Peaks obtained with cellosyl, used as a control of PG muropeptide composition, are numbered as in Bui *et al.* 2012^7^. Corresponding structures of the LytB-released fragments (marked with apostrophes) have similar retention time but differ in the sugar at the reducing end (*N*-acetylglucosaminitol for LytB vs. *N*-acetylmuramitol for cellosyl; see Supplementary Fig. S4). PG fragment structures are shown in Supplementary Fig. S3. Labels with asterisks indicate deacetylated muropeptides and those underlined correspond to peaks analyzed by MS (see Supplementary Table S2). The structures corresponding to C1-C2 peaks could not be determined due to the co-elution of phosphate from the sample buffer, which interferes with ESI-MS/MS analysis.

LytB substrate specificity was investigated by analyzing the products released by digestion of purified cell walls from pneumococcal strains (R36A, R36A::*pgdA*, R6B, Pen6, Pen6*adr* and CS1) whose particular characteristics are detailed in Table 1. As control, assays were run in parallel with PG cleavage by the muramidase cellosyl, which completely digests the pneumococcal PG and, consequently, was previously used to analyse its composition^7^. The results are summarized in Fig. 3, Fig. S3 and Table 2 (peaks were numbered as in Bui *et al.*, 2012^7^). As expected, LytB products differed from cellosyl products in that they contained GlcNAc and not MurNAc at the reducing end, confirming that LytB is an *N*-acetylglucosaminidase (Supplementary Fig. S4). Interestingly, there were three other major differences between the products generated by the two enzymes. First, *N*-deacetylated species were not detected in the LytB products, but were present, as expected, in those of cellosyl that released Tri[deAc], Tetra(S)[Glu/deAc], TetraTri[deAc]^‡^ and TetraTetraTri[deAc]_2_[-GM] (peaks 2, 10, 17/18, and 29, respectively) among other deacetylated fragments upon digestion of PG from the R36A strain. Second, LytB showed a preference for uncross-linked muropeptides (monomers) (peaks 3′–16′) and the TetraTri dimer (peak 19′), whereas dimers with Ser-Ala or Ala-Ala bridges, and even trimers, represented a higher percentage of the reaction products in the hydrolysis with cellosyl (peaks 23, 25, 39 and 31). Third, LytB release of early eluting, putative non-substituted glycan chains (fractions C_1_ to C_2_), was around 10 times higher than with cellosyl (Table 2) and markedly increased in R6B lysates. Accordingly, digestion of cell walls or PG from R36A::*pgdA* containing full acetylation of GlcNAc moieties resulted in more similar profiles by raising the relative contribution of Tri, TetraTri and Tetra(SA)Tri muropeptides (peaks 3′, 19′ and 23′, respectively) in LytB products, and the amount of material solubilised by the pneumococcal glucosaminidase was almost doubled with respect to the R36A strain (~66% of that of the cellosyl digest for the R36A::*pgdA* strain). Furthermore, LytB and cellosyl solubilised to a similar extent the branched Tri(SA) and Tri(AA) monomers (fractions 12/12′ and 14/14′, respectively), which are enriched in strain Pen6 and Pen6*adr*, but LytB failed to efficiently release the branched dimers or trimers (the cellosyl products 29, and 31). Cross-linked muropeptides became almost undetectable also in the reaction products of LytB with the cell wall of the pentapeptide-rich CS1 mutant (Fig. 3 and Table 2), but not for cellosyl, and the Penta muropeptide (peaks 7/7′) became the predominant species. Of note, the amount of cell walls solubilised by LytB was ~50% higher for the CS1 mutant than for R36A. The lower abundance of putative unsubstituted glycan chains in cellosyl digests could be explained, at least partially, by the overall more material released by this enzyme. Hence, these data show that LytB cleaves PG at sites with fully acetylated GlcNAc residues with preference for monomeric peptides, with or without branches, and is less active at sites with peptide cross-links. Moreover, *in vitro* degradation of cell walls labelled with [*methyl*-^3^H]choline showed that prior cleavage of peptide stems by the LytA amidase increased the specific activity of LytB by around 4-fold (from 600 ± 60 U/mg to 2,500 ± 600 U/mg; average of five independent experiments), indicating that removal of the stem peptides, or the resulting increase in size of the cell wall pores, facilitated the catalytic action of LytB. This activity is lower than that of LytA (~2.5 ×10^5^ U/mg) but in the order of that of LytC (~6,000 U/mg)^13^.

### Cleavage of PG structural analogues

**Figure 4:**
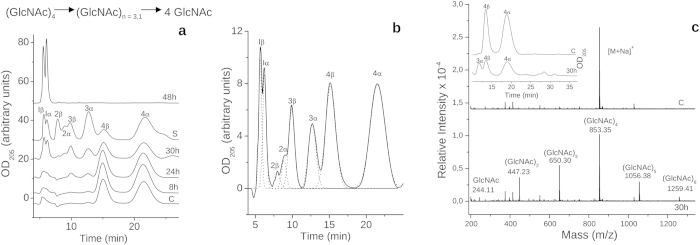
HPLC and MALDI-TOF analysis of (GlcNAc)_4_ products upon treatment with LytB. The reaction was carried out at 37 °C in Pi buffer, pH 7.0. Aliquots of the reaction mixture were withdrawn at different incubation times and compounds were detected following the absorbance at 205 nm (arbitrary units). (**a**) Incubation of (GlcNAc)_4_ (84 μM) with LytB (4.2 μM). The substrate and products formed during the reaction are shown at the top. (**b**) Deconvolution of the elution profile after 30 h of incubation from panel (**a**). (**c**) Detection of transglycosilation products by HPLC and MALDI-TOF after 30 h of incubation of (GlcNAc)_4_ (1.5 mM) with LytB (4 μM). Product masses (*m/z*) correspond to the [M + Na]^+^ species of (GlcNAc)_5_ and (GlcNAc)_6_ together with the hydrolysis products. Monoisotopic masses [M + Na]^+^ are presented for each of the compounds detected. Controls without protein at the longest incubation time are shown (C traces). Peak labels indicate the number of GlcNAc units and the anomeric form (α or β) of each species, assigned by using GlcNAc oligomers (n = 1–6; 300 μM each) as standards (S trace).

The complexity of cell-wall hydrolysis by LytB abrogates its use in investigation of the reaction mechanism. With this aim in mind we tested the capacity of LytB to cleave small compound surrogates of PG components (see Supplementary Fig. S5). First, the *N*-acetylglucosamine oligomers (GlcNAc)_4_ and (GlcNAc)_5_ were used as substrate because of LytB structural relationship with chitinases and lysozymes with chitin hydrolase activity^34^. LytB cleaved both oligosaccharides but products were only detected after several hours of incubation (Fig. 4 and Supplementary Fig. S6), indicating that productive-complex formation required a slow rearrangement of either the free protein or previously formed non-productive complexes^29^. Subsequently, the reaction advanced relatively rapidly and all possible digestion intermediates were identified using HPLC and MALDI-TOF yielding, with both substrates, GlcNAc as final product (Fig. 4 and Supplementary Fig. S6). Nevertheless, after 30 h of incubation GlcNAc and (GlcNAc)_3_ or (GlcNAc)_4_ were the principal products of (GlcNAc)_4_ or (GlcNAc)_5_ hydrolysis, which indicated preferential occupancy of subsites −1 to +(n−1) and/or −(n−1) to +1 by the substrate. GlcNAc oligomers were also hydrolysed by the D657A protein mutant, albeit at lower rate than by the wild-type enzyme, as shown for (GlcNAc)_4_ in Supplementary Fig. S7, in agreement with the effect of this mutation on cell chain dispersing activity.

Of note, several new peaks eluting after that of the initial substrate were observed in the presence of LytB, their molecular masses being compatible with the incorporation of one or two GlcNAc units to the initial substrate (Fig. 4c and Supplementary Fig. S6). The simplest interpretation of these results is that LytB can also catalyze the glycosyltransfer reaction, though less efficiently than the hydrolysis considering the relative intensity of respective products. This activity, also reported for certain derivatives of HEWL and T4 lysozymes^35^,^36^, has only been observed in retaining enzymes^32^. Future investigations will shed light if the same applied to LytB.

The cleavage of small muropeptides was also tested using GlcNAc-MurNAc-L-Ala-D-γ-Gln (GMDP), GlcNAc-MurNAc(OCH_3_)-L-Ala-D-γ-Glu-L-Lys-D-Ala-D-Ala (GMPP) and GlcNAc-MurNAc-GlcNAc-MurNAc(OCH_3_) (GM)_2_ as substrates. The analysis of the reaction mixtures revealed that only the tetrasaccharide is recognized as substrate by LytB (Supplementary Figs. S8 and S9), yielding GlcNAc-MurNAc-GlcNAc and MurNAc(OCH_3_) as the sole reaction products, as confirmed by LC/MS and LC/MS/MS (Supplementary Fig. S9). Conversion was very slow (~10% turnover in 48 h) but clearly documents that the tetrasaccharide is the smallest fragment of the PG glycosidic chain recognised as substrate by LytB, that the tetrasaccharide occupies subsites −3 to +1, and that cleavage corresponds to an unequivocal glucosaminidase reaction. The poor turnover rate of LytB of this substrate might be due to the presence of the negatively charged carboxylates of the MurNAc residues.

### Definition of substrate-binding subsites and modelling of PG binding to LytB_CAT_

The best structural agreement between LytB_GH73_ and the related structures of the GH23 family (the closest relatives identified by the Dali server^37^) corresponds to the region defining the substrate-binding groove (helices α-3 and α-5), including the proton-donor and the aromatic residues that help to create an environment that favours catalysis at physiological pH^17^,^19^,^20^,^21^,^28^ (Supplementary Fig. S10). Moreover, superimposition of LytB_GH73_ with the related GH23 structures containing substrates or inhibitors at the active site revealed a good fit of the ligands into the substrate-binding groove of LytB (Supplementary Fig. S10) and allowed identification of at least 5 different subsites in its catalytic cavity, from positions −3 to +2 (the reducing end), with the saccharide units flanking the scissile bond (positions −1 and +1) close to Glu585, the proposed proton donor. The saccharide unit at position −4 in GH23 complexes would be out of the LytB_GH73_ catalytic groove.

**Figure 5:**
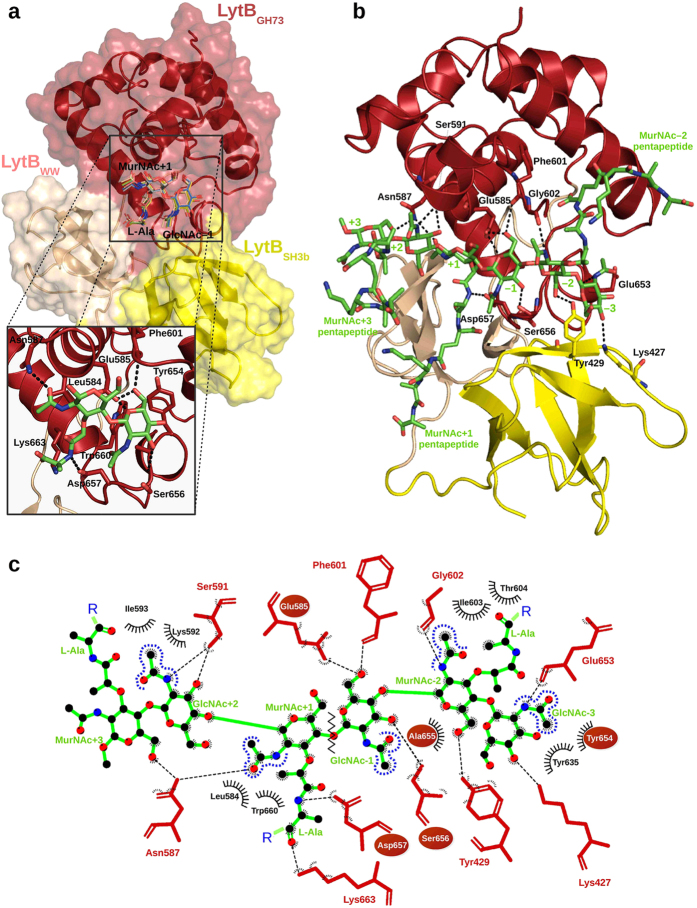
Models of LytB_CAT_ complexes with PG analogues. (**a**) Surface representation of the LytB_CAT_/GlcNAc-MurNAc-L-Ala complex generated by AutoDock 4.2. Saccharide units are docked into +1/−1 subsites of the binding site. The ten lower energy solutions obtained are drawn in line representation. The inset shows the contact network at subsites +1 and −1 for the best docking complex. Dashed lines indicate the hydrogen bonds and the residues involved are shown as red (LytB_CAT_) or green (ligand) sticks. (**b**) Putative interactions between the domains of LytB_CAT_ and (GMPP)_3_ (green sticks) in the computational model created by extending the best docking solution for the LytB_CAT_/GlcNAc-MurNAc-L-Ala complex. Protein residues involved in hydrogen bond formation are depicted as sticks. (**c**) Schematic representation of contacts between LytB_CAT_ and (GMPP)_3_ glycan strand analyzed with LigPlot+ v.1.4^65^. Van der Waals interactions are shown with black arcs. The catalytic Glu585 and the conserved YASD tetrad are highlighted with red ovals, and the zig zag line indicates the scissile bond. The same colour code was used in the whole figure: GlcNAc-MurNAc-L-Ala and (GMPP)_3_ are depicted in green, whereas LytB_GH73_, LytB_SH3b_ and LytB_WW_ domains are in red, yellow and salmon, respectively.

To provide a basis for better understanding of the substrate recognition at the molecular level we performed docking studies using first GlcNAc-MurNAc-L-Ala as ligand. The 10 low-energy complexes found by AutoDock for GlcNAc-MurNAc-L-Ala bound to LytB_CAT_ placed the GlcNAc and MurNAc moieties at subsites −1 and +1 of GH23 complexes (hereafter GlcNAc−1 and MurNAc+1; see Fig. 5a). In the best-scoring energy pose of the cluster, the carboxylate oxygen of Glu585 is at hydrogen-bond distance of GlcNAc−1 O5 and O6 hydroxyl oxygens and at 4.0 Å from the anomeric oxygen, with the suitable orientation for involvement in catalysis. Two additional hydrogen bonds by Ser656 and Phe601 contribute to GlcNAc**−**1 recognition. The *N*-acetyl group of MurNAc+1 also established hydrophobic and polar interactions with Leu584 and Asn587, respectively, whereas the lactyl moiety makes hydrophobic contacts with Lys663 and Trp660 residues, and the side-chain of Asp657 (relevant for catalysis) is bridged to the attached L-Ala. Equivalent contacts have been described for the sugar unit at position −1^38^,^39^ and the *N*-acetyl group of the sugar unit at position +1^40^,^41^ in several complexes of GH23 family. The partially conserved, intricate network of substrate-protein interactions here predicted for the GlcNAc-MurNAc-L-Ala/LytB_CM_ complex supports the notion that our model is plausible.

Subsequently, a larger PG structure was modelled into the substrate-binding cavity by double superimposition of the tetrasaccharide di-pentapetide GlcNAc-MurNAc(-L-Ala-D-γ-Glu-L-Lys-D-Ala-D-Ala)-GlcNAc-MurNAc(-OCH_3_)(-L-Ala-γ-D-Glu-L-Lys-D-Ala-D-Ala) ((GMPP)_2_) structure onto the GlcNAc-MurNAc-L-Ala/LytB_CAT_ complex. The final model revealed contacts for the six saccharide units occupying the binding cleft (from positions −3 to +3), but the MurNAc unit at subsite +3 was nearly out of the cavity (Fig. 5b,c). The glycan strand retained the helical conformation found in solution for (GMPP)_2_^42^, MurNAc + 1 and GlcNAc−1 keep the orientation and contacts found in the previous model, and the saccharide units at subsites +2, −2 and −3 were docked as expected from the superimposition of LytB_CAT_ with the GH23 complexes shown in Supplementary Fig. S9. Contact inspection showed that the glycan strand is hydrogen-bonded to 13 functionalities from 10 different residues (Table S3) and make about 10 favourable hydrophobic interactions (Fig. 5b,c), a high proportion of which are concentrated at subsites +1 to −2. Thus, MurNAc−2 is involved in polar or hydrophobic interactions with several residues from the LytB_SH3b_ (Tyr429) and LytB_GH73_ (Gly602, Ile603 and Thr604) domains, largely mediated by the sugar *N*-acetyl group (Fig. 5c). Relevant contacts at subsites +2 and −3 further document the importance of *N*-acetylation for the PG recognition by LytB. Thus, the *N*-acetyl group of GlcNAc+2 interacts with a number of residues from helix *α*-4, while that of GlcNAc−3 can mediate contacts with residues close to the conserved YASD tetrad of the GH73 family (Fig. 5b,c and Table S3). Atoms from the main chain and the side chains of the pentapeptides bound to MurNAc+1 and MurNAc−2 would also make favourable contacts with several protein residues (Fig. 5b and Table S3), but that of MurNAc+3 appears to be completely exposed to the solvent. The hydrogen bonds mediated by the MurNAc+1 pentapeptide, docked at the interface of LytB_SH3b_ and LytB_WW_ domains, expand to side chains of residues belonging to the three domains of LytB_CAT_, whereas the MurNAc−2 pentapeptide, laying within a hydrophobic cavity of LytB_GH73_ limited by helix α-5 and the flexible segment connecting α-4 to α-5, could bind through hydrogen bonds and hydrophobic contacts to residues of helices *α*-3 and *α*-5 and the loop connecting *α*-4 to *α*-5 (see Supplementary Fig. S11 and Table S3).

### Determinants of LytB cellular localization

**Figure 6:**
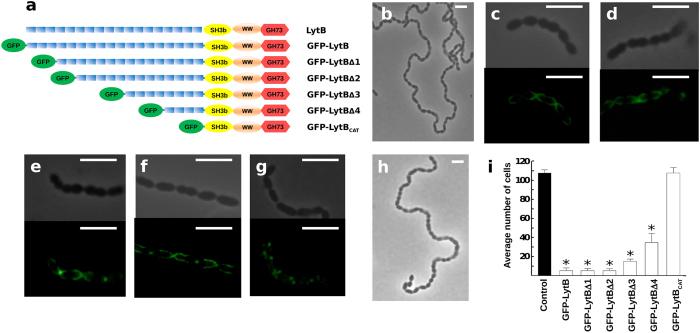
Localization of different GFP-LytB deletion proteins at the surface of *S. pneumoniae* and their dispersing chain activity. (**a**) Scheme of LytB deletion mutants fused to the GFP. Symbols have the same meaning as in Fig. 1a. (**b**–**h**) R6B cultures (OD_550_ ~ 0.15) were treated at 37 °C with the purified proteins (9 μg/ml) and observed at the microscope, with or without fluorescence, after 30 min of incubation. (**b**) control of untreated cell culture (**c**) GFP-LytB; (**d**) GFP-LytBΔ1; (**e**) GFP-LytBΔ2; (**f**) GFP-LytBΔ3; (**g**) GFPLytBΔ4; (**h**) GFP-LytB_CAT_. (**i**) Average chain length of R6B cells after 30 min of incubation with the different proteins. Error bars represent standard deviations, and asterisks mark results statistically significant compared to the control in the absence of protein (one-way ANOVA with a *post hoc* Dunnet test; *P < 0.01). Bars, 4 μm.

As LytB harbours the highest number of CBRs among the pneumococcal choline-binding proteins (18 repeats in LytB from R6 strain) and choline plays a key role in the bacterial physiology and the enzymatic activity of PG hydrolases, we investigated the participation of the CBRs in LytB cellular localization and activity. In this vein, recombinant genes coding for chimeric proteins containing the green-fluorescent protein (GFP) fused to the full-length protein or serial truncated mutants with 15, 13, 8 and 4 CBRs or just LytB_CAT_ (Fig. 6a) were cloned and the corresponding proteins purified to electrophoretic homogeneity. Each LytB variant was exogenously added to an R6B culture to test both its localization on pneumococcal cells and its capacity to disperse the long cell chains, using the untreated culture as control (Fig. 6b). These experiments show that the fluorescence signals of full-length GFP-LytB and truncated GFP-LytBΔ1, GFP-LytBΔ2, GFP-LytBΔ3 and GFP-LytBΔ4 proteins (with 18, 15, 13, 8 and 4 CBRs, respectively) have a preferential polar localization, although dispersed localization over the cell hemispheres was also observed (Fig. 6c–g). However, the cell de-chaining capacity of these constructions depended on the length of the LytB_CBM_: GFP-LytB, GFP-LytBΔ1 and GFP-LytBΔ2 displayed the same chain-dispersing activity, while that of GFP-LytBΔ3 was slightly reduced and GFP-LytBΔ4 moderately decreased the length of cell chains (Fig. 6i). In contrast, no binding nor dispersion was detected with the GFP-LytB_CAT_ protein lacking all the CBRs (Fig. 6h,i). Bai *et al.*, (2014) have previously reported that LytB_CAT_ could disperse cell chains of a TIGR4 *lytB* mutant^17^, which might be due to the moderate cell chain length of this mutant and the use of 10-fold higher protein concentration in their assays. It is noteworthy that LytB deletion variants without GFP had the same chain-dispersing capacity than the corresponding GFP-constructions, confirming that the N-terminal GFP tag did not interfere with the catalytic activity.

**Figure 7:**
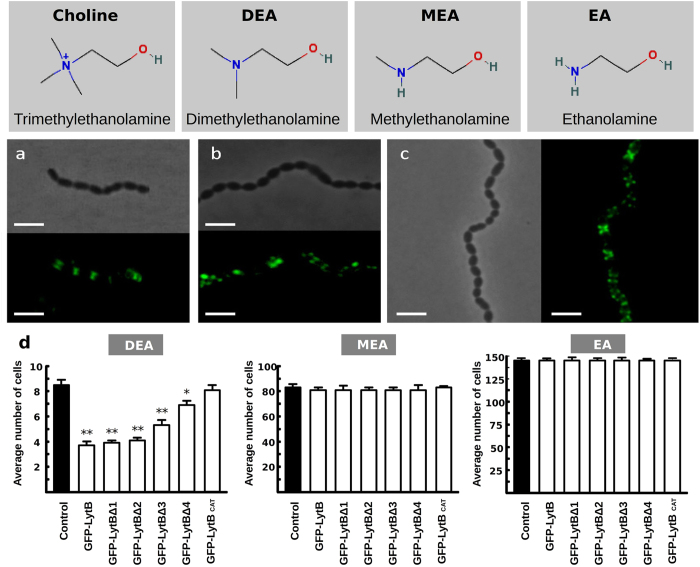
Influence of the amino alcohol incorporated in the (lipo)TAs on the localization and chain dispersing activity of different LytB protein variants. Top panels, structures of choline and the tested analogs. Middle panels, fluorescent signals of R6 cultures grown in media with different amino alcohols taken after 30 min of incubation with GFP-LytBΔ1 protein. (**a**) Culture in DEA-containing medium; (**b**) Culture in MEA-containing medium; (**c**) Culture in EA-containing medium. Bottom panels, average chain length of DEA, MEA or EA cells after 30 min of incubation at 37 °C with the different LytB constructions. Error bars and asterisks mean as in Fig. 6 (*P < 0.05; **P < 0.01). Bars, 4 μm.

The dependence of LytB binding and activity on the aminoalcohol incorporated in TAs was also examined using the R6 wild-type strain grown in chemically defined media containing either choline or its chemical analogues *N*-*N*-dimethylethanolamine (DEA), *N*-methylethanolamine (MEA) or ethanolamine (EA). As shown in Fig. 7a–c, the GFP-LytBΔ1 protein binds to defined zones of all types of cells with low spreading of the fluorescent signal over the cell hemispheres. The cell-dispersing activity of GFP-LytB and the truncated constructs was checked also on the same DEA-, MEA- and EA-grown cells. As shown in Fig. 7d, the GFP-fusion proteins displayed the same activity graduation seen on choline-grown cultures when tested on DEA cells (*i.e.* a partial decrease of the chain length that increased with the LytB_CBD_ length) and no de-chaining activity on MEA and EA cells. Taken together, these experiments revealed that (i) the exogenously added protein is primarily located on polar regions, with similar patterns for constructs containing from 18 to 4 CBRs, although the activity of the mutant with only 4 CBRs is significantly impaired; (ii) binding to and localization on pneumococci do not depend on the nature of the amino-alcohol decorating the bacterial TAs but requires the presence of the CBRs; and (iii) the dispersing activity was totally dependent on the presence of choline — or a tertiary-aminoalcohol — in the culture media.

## Discussion

Typical pneumococci grow as diplococcal or short chained cells and these forms are governed by the correct function of the LytB glucosaminidase. The direct consequence of *lytB* gene knockout is the formation of long cell chains^13^,^16^,^17^,^26^, but also impairments to biofilm formation^18^,^43^, respiratory track colonization and infection^14^,^15^,^17^, and evasion of host immunity^15^, although the precise effect of *lytB* gene inactivation might depend on serotype and growth conditions^13^,^16^,^17^,^26^. Separation of daughter cells entails cleavage of the glycosidic bonds of PG backbone units, and full integrity and activity of LytB is also important for lung epithelial cell colonization and infection^17^. We have documented that LytB is a cell-wall glucosaminidase that can also hydrolyse soluble GlcNAc oligomers and displays a small, but detectable, glycosyltransferase activity, but the physiological relevance of these new activities is not yet clear. However, considering that the pneumococcal biofilm matrix contains a polysaccharide with *β*-(1,4)-GlcNAc units and is highly sensitive to the action of chitinases^41^, these activities might be related to enhanced biofilm formation at low concentrations of LytB (≤ 0.5 μg/ml) and/or the ability to disintegrate biofilms at concentrations ≥5 μg/ml^41^. The existence of cell-associated LytB−eDNA complexes in *S. pneumoniae* biofilms has been also reported^44^.

The failure of LytB to hydrolyze the smaller muropeptides GMDP and GMPP could denote the importance of individual binding sites for natural PG recognition and, consequently, a non-productive mode of binding^29^. Moreover, the (GM)_2_ tetrasaccharide is shown to be the smallest PG fragment hydrolyzed by LytB. Besides, its cleavage pattern unveils preferential binding to positions −3 to +1. We also noted that the trisaccharide that is generated from turnover of (GM)_2_ is not a substrate for the enzyme, which underscores the importance of occupation of these subsites for the catalytic competence of the enzyme. When compared with (GlcNAc)_4_ or (GlcNAc)_5_ hydrolysis reactions, these results also denote that the lactyl moieties of (GM)_2_ determine the feasible positioning in the active site.

Modelling of the (GMPP)_3_ muropeptide in complex with LytB_CAT_ has provided a first insight into the subsite structure of the catalytic cavity and the respective ligand−protein contacts. According to the model, the binding groove could accommodate up to six saccharide units (subsites −3 to +3) and the entire pentapeptides of MurNAc-2 and MurNAc+1. Interactions between LytB_CAT_ and MurNAc+3 are apparently minor, but its recognition by the LytB_CBD_ domain cannot be dismissed. Protein contacts are primarily provided by the LytB_GH73_ domain, but the saccharide units at subsites −3 and −2 are also stabilized by residues from the LytB_SH3b_ domain, which participates together with LytB_WW_ in recognition of the MurNAc+1 pentapeptide. These observations agree with the relevance of the last three domains of LytB for the PG hydrolase activity^17^, and its capacity to efficiently hydrolyze Tri, Tetra and Penta monomers. The carboxylic oxygen of the proton donor (Glu585) is located at 4.0 Å from the anomeric oxygen of GlcNAc−1 and at 7.9 Å from the carboxylic group of Asp657, the only other acidic residue relevant for the activity, which in turn is at hydrogen-bond distance of the main-chain amine of L-Ala of the MurNAc+1 pentapeptide.

Identification of the soluble reaction products after cell-wall digestion by LytB, and their comparison with those released in parallel by cellosyl, have shown that the glucosaminidase is highly specific for fully *N*-acetylated substrates, and preferentially cleaves uncross-linked muropeptides (with and without a dipeptide branch) or directly cross-linked TetraTri dimers. Accordingly, the amount of material released upon digestion of R36A*::pgdA* fully-acetylated cell-walls was almost doubled compared to R36A, with a clear increase in the proportion of fully acetylated TetraTri and Tetra(SA)Tri muropeptides. The differential distribution of GlcN residues in the pneumococcal PG (45.1% in dimeric muropeptides *vs* 18.5% in monomers and 14.2% in trimers^7^) could partially explain the higher resistance of dimers to LytB hydrolysis in deacetylated strains but not of trimers. The flexible region of LytB_GH73_ (not visible in the crystal structure and shorter than those of Auto and FlgJ glucosaminidases) or the LytB_CBD_ domain, whose positioning in the overall protein structure remains unknown, might hamper binding of trimers or peptide-bridged-branched dimers that were almost undetected in the reaction products of all the cell walls tested. On the other hand, unsubstituted glycan chains, susceptible to LytB action, might be enriched in the thin filaments of cell wall holding together the daughter cells, as suggested its higher proportion in cell walls of R6B strain. It is worth noting that LytB was completely unable to disperse the long chains formed by a PBP2b depletion mutant whose PG was enriched in branched cross-linked muropeptides with respect to that of wild-type cells^45^. Interestingly, the significant decrease in adhesion to and invasion into lung epithelial cells caused by mutation of the catalytic proton donor to glutamine^17^, might show a correlation between virulence and LytB substrate specificity. In addition, pneumolysin release from cell-wall compartment is controlled by the increase of branched cross-linked muropeptides and depends, at least partially, on PG remodelling by non-lytic choline-binding hydrolases^46^,^47^, which makes it tempting to speculate that LytB might contribute to pneumolysin release into the surrounding medium.

At the molecular level, two different factors would account for the resistance of deacetylated substrates to LytB catalysis: (i) a direct participation of the *N*-acetyl moiety of GlcNAc−1 in catalysis, acting as the nucleophile through a substrate-assisted mechanism, and/or (ii) a key role of the *N*-acetyl moieties of GlcNAc in substrate recognition by the active site. The finding of a single acidic residue, Glu585, essential for the activity strongly indicates that the LytB-mediated hydrolysis might take place via a substrate-assisted catalysis, with Glu585 acting as the general acid and the carbonyl oxygen of the *N*-acetyl group of GlcNAc−1 as the nucleophile, thereby accounting for its critical role in activity. Nonetheless, according to the structure proposed for the LytB_CAT_/(GMPP)_3_ complex, the *N*-acetyl moieties of the GlcNAc units bound at subsites −3 and +2 are also involved in binding and would further contribute to the selectivity of PG recognition. In this context, the predicted participation of Asp657 in substrate binding would explain the slower cell dispersion observed for the D657A mutant. We further note that any of these interactions, even those remote from the seat of reaction, could contribute to solvation of the substrate within the active site. The immediate consequence of this could be manifested in lowering of the energy barrier for the critical transition-state species for the reaction catalysed by LytB.

The selectivity of LytB for fully acetylated substrates does not seem to be inherent to the entire family GH73. Though to a lesser degree, the glucosaminidase domain of the Auto autolysin from *L. monocytogenes* also cleaves deacetylated muropeptides^19^, which rules out a substrate-assisted mechanism in this GH73 family member and correlates with the identification of two carboxylates essential for the activity: Glu122 (the general acid) and Glu156 (the general base). These two residues, located near the C-terminus of helix *α*-3 and the first strand of the *β*-lobe, respectively, are 13.3 Å apart and substrate binding must promote an important structural change to get them close enough to allow for the proposed inverting mechanism^19^. The same scenario is foreseen for the FlgJ proteins from *Sphingomonas sp.* and *Salmonella enterica*, and the TM0633 protein from *Thermotoga maritima*, where two acidic residues located at equivalent positions have also been reported to be essential for the activity^21^,^28^,^30^. In contrast, the glucosaminidase module (glu_atlwm_) of the Atl_WM_ autolysin, like LytB, does not have a second acidic residue essential for catalysis in the *β*-lobe, and substitution of Asp1335 (equivalent to Asp657 of LytB) by alanine or asparagine had a minor impact on the activity^27^. These data support the notion that GH73 glycosyl hydrolases do not share a unique catalytic mechanism, and evolution towards inverting or substrate-assisted enzymes might be related to the length and/or the structure of the *β*-lobe that helps to shape the catalytic cavity^22^, thereby facilitating a fine-tuning of target substrates.

LytB acts exclusively at the poles of the cells, both endogenously and exogenously. Our results revealed, however, that LytB can solubilize as much as 36% of the PG when fragments of deacetylated cell walls are used as substrate, in agreement with previous findings^13^, and that its activity on cell walls pretreated with LytA compared with that of LytC. This raises the possibility that LytB could potentially act as an autolytic enzyme whose activity should be strictly controlled to prevent the lysis of the cell. Indeed, the failure to clone the gene fragment encoding the full-length form of LytB (with the signal peptide) in *Escherichia coli* is consistent with the notion that the unregulated mature protein, when located on the outer surface of the cytoplasmic membrane, could cause the lysis of the culture, as was shown in the cases of endolysins of pneumococcal bacteriophages EJ-1 and Cpl-1^13^. However, more evidence is needed to ascertain whether or not LytB might have the capacity to act as an autolytic enzyme, a role so far assigned to LytA and LytC. Our results indicate that LytB activity is controlled by at least three different mechanisms including: (i) choline-independent physical confinement at the cell pole regions; (ii) attachment to choline moieties of (lipo)TAs through the LytB_CBD_ domain; and (iii) PG composition (selective control of susceptible bonds through deacetylation of the GlcNAc moieties and PG cross-linking).

The differences found in the electron density of lateral walls with respect to the cell poles suggested that they are structurally and/or chemically distinct^13^. Therefore, preferential location of LytB around the poles could rely on the specific recognition of an unevenly distributed/accessible targets facilitated by LytB_CBD_, as no binding of GFP-LytB_CAT_ to R6B cells was detected. Moreover, the PG itself could behave as a selective, irregular structural entity where differences in pore dimensions might determine LytB localization or the target accession, considering the size and probable asymmetry of the overall protein structure. It is also plausible that certain components of the bacterial envelope, like (lipo)TAs or surface proteins, could cooperate to shape the selectivity of LytB by masking susceptible bonds *in vivo*. As in other choline-binding proteins^22^,^23^, the LytB_CBD_ probably provides a strong binding to the bacterial surface through multiple noncovalent bonds, thereby impairing LytB transit across the PG matrix. Elucidation of the overall protein co-structure with cell wall fragments would be needed to know if LytB_CBD_ contributes to the substrate specificity or to the proper orientation of the PG chain within the catalytic cavity.

## Methods

### Bacterial strains and growth conditions

The bacterial strains, plasmids and oligonucleotides used in this study are listed in Table 1 and Supplementary Table S1, respectively. *S. pneumoniae* was grown at 37 °C in C medium supplemented with yeast extract (0.8 mg/ml; Difco Laboratories; C + Y medium) without shaking^48^. Bacteria with TAs containing the various amino alcohol residues (such as choline, EA, MEA, or DEA) were grown for more than 20 generations in Cden medium supplemented with the appropriate amino alcohol^49^. *E. coli* was grown in Luria-Bertani (LB) medium at 37 °C with shaking.

### Cloning, expression and purification of proteins

Routine DNA manipulations were performed essentially as described previously^50^. To construct the single alanine mutants of LytB (Glu585, Asp596, Asp607, Asp618, Asp619, Asp621, Glu633, Asp637, Glu653, Asp657, Glu662 and Glu673) appropriate oligonucleotides were used (Supplementary Table S1). Site-directed mutagenesis were performed with Pfu DNA polymerase (Biotools) according to the manufacturer’s instructions^51^ using the plasmid pRGR5^13^ as template, and the recombinant plasmids were transformed into *E. coli* BL21(DE3). In addition, relevant fragments for construction of deletion mutants were cloned into the pT7-7 plasmid^52^ using *Nde*I and *Pst*I for constructions without GFP, or *Bam*HI and *Pst*I for fusion proteins with the GFP, and the resulting recombinant plasmids were transformed into *E. coli* BL21(DE3). The accuracy of all cloned genes was confirmed by DNA sequencing (Secugen S.L.).

For protein overproduction, transformed *E. coli* cells were incubated in LB medium containing ampicillin (0.1 mg/ml) to an optical density at 600 nm (OD_600_) of about 0.6. Isopropyl-β-D-thiogalactopyranoside (0.1 mM) was then added and incubation was continued overnight at 24 °C. Cells were harvested by centrifugation (10,000 × *g*, 10 min), resuspended in 20 mM sodium phosphate buffer (hereafter Pi buffer), pH 6.9, and disrupted in a French pressure cell. Typically, insoluble fractions were separated by centrifugation (15,000 × *g*, 20 min), and the supernatants were loaded onto a DEAE-cellulose column for standard purification, in a single step, according to the procedure described for choline-binding proteins^53^. Additionally, LytB wild type and the alanine mutants were subjected to a size-exclusion chromatography on dextran-agarose (Superdex-200, GE Healthcare) to remove possible aggregates or minor components of lower molecular weight. Column pre-equilibration and protein elution was carried out with 20 mM Pi buffer, pH 8.0, with 140 mM choline. GFP-LytBΔ4 and GFP-LytB_CAT_ proteins, with four and none CBRs, respectively, were purified by chromatography on DEAE-cellulose using a 0.5–1.5 M NaCl gradient for elution. Protein concentrations were determined spectrophotometrically using the molar absorption coefficients at 280 nm. Before use, proteins were dialyzed at 4 °C against the appropriate buffer and centrifuged for 5 min at 11,600 × *g.*

### Isolation of pneumococcal cell wall and PG

*S. pneumoniae* laboratory strains R36A, the penicillin-resistant strains Pen6, the attenuated variant Pen6*adr*, and the mutant strains R6B, R36A::*pgdA*, and CS1 (Table 1) were grown to an OD_620_ of 0.5 in C + Y medium. Cell walls and purified PG from these strains were prepared as described^7^.

### Chemicals

Oligonucleotides were purchased from IDT (Integrated DNA Technologies, Inc) or Sigma. The muramidase cellosyl was a gift of Hoechst (Frankfurt, Germany). (GlcNAc)_n_ derivatives (n = 1 to 6) were from Toronto Research (Canada). GMDP was from Merck, and GMPP and (GM)_2_ were synthesized as described^54^,^55^.

### Chain-dispersing activity assay

Chain dispersing capacity of LytB variants was assayed on pneumococcal R6B cells, deficient in LytB. Cultures were grown up to an OD_550_ of 0.2 and then treated with the enzyme (10 μg/ml) at 37 °C. Aliquots were removed at different incubation times (0.5 to 5 h) and examined by phase-contrast microscopy (Leica DM4000B; Microsystems); the average cell number in chains was also determined by counting at least 50 separate chains of cells from several independent experiments. Control samples contained Pi buffer (pH 6.9) instead of LytB. Each value represents the mean ± SD of 3 to 4 replicates. Statistical analysis was performed by using analysis of variance (ANOVA) for multiple comparisons. GraphPad InStat version 5.0 (GraphPad Software, San Diego, CA) was used for statistical analysis.

### *In vitro* cell-wall activity assay

The cell wall degradation activity of LytB was quantitatively assayed at 37 °C in Pi buffer (pH 7.0) using [*methyl*-^3^H]choline-labeled pneumococcal cell walls from strain R6 as substrate^56^, following the protocol described elsewhere^57^. To test the effect of peptide stem removal on LytB activity, cell walls (20 μl) were pre-treated for 15 min at 37 °C with low concentrations of LytA amidase (6.0–250 ng/ml). The reaction was stopped by heating for 5 min at 100 °C and samples were centrifuged at 4 °C (2 min, 10,000 × *g*) before treating with LytB (23 μg/ml). The mixture was incubated at 37 °C for further 15 min and LytB activity was determined by comparing the radioactivity in the supernatant with controls where the LytA amidase or LytB were substituted by buffer during the first and second incubation rounds, respectively. One unit of activity (U) was defined as the amount of enzyme that catalyses the hydrolysis (solubilization) of 1 μg of cell wall material in 10 min.

### Characterization of cell-wall hydrolysis products

Muropeptides released from the pneumococcal cell walls by LytB were reduced and analyzed by HPLC following a previously published procedure^7^ with certain modifications. In parallel, PG was digested with the muramidase cellosyl that is used to determine muropeptide composition^7^. PG suspensions (~0.25 mg in 200 μl) were stirred with 10 μg of cellosyl in 20 mM Pi buffer (pH 4.8), and cell walls (~0.40 mg in 200 μl) with 20 μg of LytB in Pi buffer (pH 7.0) supplemented with 100 mM sodium chloride. After 24 h at 37 °C new enzyme was added, and the hydrolysis proceeded for another 24 h. Reactions were stopped by either heating 10 min at 100 °C (cellosyl) or cooling at 4 °C (LytB). Samples were then processed and separated on a reverse-phase column (Prontosil 120-3-C18-AQ, 250 × 4.6 mm, 3 μm, Bischoff, Germany) as described^7^. The total peak area (excluding the salt fractions eluting before 8 min) was normalized to 100%, and the relative area of the products determined. Selected fractions were collected for electrospray ionization-tandem mass spectrometry analysis (ESI-MS/MS) at the Newcastle University Pinnacle facility using a Finnigan LTQ- FT mass spectrometer^58^.

### Cleavage of PG structural analogues

The capacity of LytB to hydrolyze GlcNAc oligomers (chitinase activity) or PG derivatives was evaluated by HPLC and mass spectrometry using (GlcNAc)_4_, (GlcNAc)_5_, GMDP, GMPP and (GM)_2_ (Supplementary Fig. S5) as potential substrates. Reaction mixtures containing LytB and the assayed compounds were incubated at 37 °C in Pi buffer, pH 7.0, using protein/substrate molar ratios of 1:20–1:375 with similar results. At selected times, aliquots were withdrawn and the reaction stopped by cooling at 4 °C (GlcNAc oligomers) or adding 20 μl of formic acid in 0.05% acetonitrile (v/v) (muropeptides). Controls without LytB were run in parallel.

Hydrolysis of (GlcNAc)_4_, (GlcNAc)_5_, GMDP and GMPP was analyzed by HPLC on a reversed-phase column (*Tracer Excel 120 ODS-B*, 250 × 4 mm, 5 μm, Teknokroma) using a *Shimadzu* LC-10AV VP instrument. Samples (35 μl) were eluted at 20 °C using a linear 30-min gradient from buffer A (50 mM sodium phosphate (pH 4.2) and 15 μM NaN_3_) to buffer B (75 mM sodium phosphate (pH 4.92) and 15% methanol) for GlcNAc oligomers, whereas for synthetic muropeptides a 40-min gradient from 2% to 15% of acetonitrile in 0.1% trifluoroacetic acid was used^59^. The flow was 0.5 ml/min and compounds were detected at 205 nm. Reaction products were also checked by MALDI-TOF mass spectrometry in a Voyager DE-PRO mass spectrometer (Applied Biosystems) in the reflector positive ion mode using the following conditions: 20–25 kV acceleration voltage, 72–94% grid voltage, 0.001–0.05% guide wire voltage, and 200–400 ns of delay time reflector^60^. The spectra were obtained over an m/z range of 100–7000 and external mass calibration was applied using des-Arg1 bradykinin and angiotensin I of Calibration Mixture 1 (Sequazyme Peptide Mass Standards Kit; Applied Biosystems).

Hydrolysis of (GM)_2_ was analysed by LC/MS on a reversed-phase column (Acquity UPLC HSS T3, 150 × 2.1 mm, 1.8 μm, Waters) using a Dionex Ultimate 3000 Rapid Separation UPLC system with a Dionex Ultimate 3000 photodiode array detector coupled with a Bruker MicrOTOF-Q II quadrupole time-of-flight hybrid mass spectrometer. Detailed LC/MS and LC/MS/MS conditions were described previously^61^. Samples were eluted at 40 °C using a linear 15-min gradient from 0% to 5% of acetonitrile in 0.1% formic acid.

### Docking studies

Docking studies were performed with the AutoDock 4.2 program^62^ using GlcNAc-MurNAc-L-Ala as a first ligand and the atomic coordinates of the LytB_CAT_ fragment deposited in the Protein Data Bank entry 4Q2W^17^. The protein was considered rigid and the ligand flexible. The GlcNAc-MurNAc-L-Ala structure was carefully built using the Pymol visualizer (PyMOL Molecular Graphics System, Version 1.5.0.4 Schrödinger, LLC) followed by molecule optimization and energy minimization using the PRODRG2 server^63^. Ligand and protein files were edited and prepared with AutoDockTools 1.5.6 program^62^. Polar hydrogens and Kollman charges were added to LytB structure. Gasteiger charges were computed for the ligand, whose active torsion angles were allowed to rotate during docking. A grid box centered in the catalytic residue Glu585 with 54 × 60 × 56 grid points spaced 0.375 Å was prepared to calculate the affinity maps of the ligand in the active site using the AutoGrid4 program. For docking simulations, 100 Lamarckian Genetic Algorithm simulation runs were performed with 25 million energy evaluations per run. The best docking solution was selected by clustering within the default 2.0 Å rmsd value, and ranking the largest cluster solutions by the free energy AutoDock scoring function. Finally, the selected ligand–receptor binding mode was processed by adding hydrogens and minimizing with the FF10^64^ and GLYCAM06^65^ force fields using the Amber12 package.

To construct the model of the hexasaccharide tripentapeptide (GMPP)_3_ in complex with LytB_CAT_, the NMR 3D structure of (GMPP)_2_^42^ (Supplementary Fig. S5) was superimposed twice onto the LytB_CAT_/GlcNAc-MurNAc-L-Ala model using PyMOL. First, the reducing-end was extended by two saccharide units via structural superimposition of the GlcNAc1-MurNAc1 units of (GMPP)_2_ onto the docked GlcNAc-MurNAc-L-Ala muropeptide, followed by ten thousand steps of steeped descent and conjugate gradient energy minimization using the Amber12 software^64^. The same procedure was then repeated, but overlapping with the GlcNAc2-MurNAc2 units of (GMPP)_2_ onto the GlcNAc-MurNAc-L-Ala ligand in order to extend the non-reducing end of the glycan strand. The two partially overlapping (GMPP)_2_ molecules were merged into a single fragment with three disaccharide pentapeptide units before performing the last minimization. The stem peptides were allowed to move freely during all minimization steps. Substitution of the D-γ-Glu moiety of (GMPP)_2_ by D-γ-Gln did not alter the complex structure.

### Miscellaneous methods

Observation of the cell morphology of pneumococci was carried out at the mid-exponential phase of growth by phase-contrast or epifluorescence microscopy with a Leica DM4000B Microsystems microscope. LigPlot+ v.1.4^66^ was used to represent the ligand binding interactions and the Dali server^37^ for 3D structural comparison.

## Additional Information

**How to cite this article**: Rico-Lastres, P. *et al.* Substrate recognition and catalysis by LytB, a pneumococcal peptidoglycan hydrolase involved in virulence. *Sci. Rep.* **5**, 16198; doi: 10.1038/srep16198 (2015).

## Supplementary Material

Supplementary Information

## Figures and Tables

**Table 1:** Bacterial strains and plasmids used in this study.

Strain	Description	Source/reference
*S. pneumoniae*		
R36A	Non-encapsulated laboratory strain	67
R36A::*pgdA*	Mutant derivative of strain R36A. Gene encoding peptidoglycan GlcNAc deacetylase is inactivated. Absence of *N*-deacetylated muropeptides	6
R6	Derivative of the rough, non-encapsulated strain R36A	68
R6B	R6 deficient mutant in *lytB* gene (R6 (*lytB*::*ermC*))	69
Pen6	High β-lactam resistance transformant enriched in branched muropeptides	70
Pen6*adr*	*Mariner* insertion in *adr* of Pen6. Enriched in branched muropeptides. Lower resistance to β-lactam antibiotics. No O-acetylation.	71
CS1	*dacA* mutant pentapeptide rich	72
*E. coli*		
BL21 (DE3)	F^−^ *bmpT gal* [*cdm*] [*lon*] *hsdS*_*b*_ with *DE3*	73
Plasmids		
pT7-7	Ap^r^	52
pRGR5	pT7-7 derivative encoding LytB wild type, Ap^r^	13
pRGR5E585A	pT7-7 derivative encoding LytB E585 mutant, Ap^r^	This study
pRGR5D596A	pT7-7 derivative encoding LytB D596A mutant, Ap^r^	This study
pRGR5D607A	pT7-7 derivative encoding LytB D607A mutant, Ap^r^	This study
pRGR5D618A	pT7-7 derivative encoding LytB D618A mutant, Ap^r^	This study
pRGR5D619A	pT7-7 derivative encoding LytB D619A mutant, Ap^r^	This study
pRGR5D621A	pT7-7 derivative encoding LytB D621A mutant, Ap^r^	This study
pRGR5E633A	pT7-7 derivative encoding LytB E633A mutant, Ap^r^	This study
pRGR5D637A	pT7-7 derivative encoding LytB D637A mutant, Ap^r^	This study
pRGR5E653A	pT7-7 derivative encoding LytB E653A mutant, Ap^r^	This study
pRGR5D657A	pT7-7 derivative encoding LytB D657A mutant, Ap^r^	This study
pRGR5E662A	pT7-7 derivative encoding LytB E662A mutant, Ap^r^	This study
pRGR5E673A	pT7-7 derivative encoding LytB E673A mutant, Ap^r^	This study
pRGR25B	pT7-7 derivative encoding fusion protein GFP-LytB, Ap^r^	13
pTRDGB1	pT7-7 derivative encoding fusion protein GFP-LytBΔ1, Ap^r^	This study
pTRDGB2	pT7-7 derivative encoding fusion protein GFP-LytBΔ2, Ap^r^	This study
pTRDGB3	pT7-7 derivative encoding fusion protein GFP-LytBΔ3, Ap^r^	This study
pTRDGB4	pT7-7 derivative encoding fusion protein GFP-LytBΔ4, Ap^r^	This study
pTRDGB5	pT7-7 derivative encoding fusion protein GFP-LytBΔ5, Ap^r^	This study

**Table 2:** Percentages of different soluble products released from the cell wall or PG by LytB or cellosyl, respectively, determined by HPLC.

Relative area (%)	*Streptococcus pneumoniae* strain					
	R6B	R36A	R36A::*pgdA*	Pen6	Pen6adr	CS1
putative glycan chains (8–24 min)						
Cellosyl	8.4	4.1	5.3	2.6	3.1	14.5
LytB	31.6	25.9	16.9	17.0	23.5	26.5
unbranched monomers (24–42 min)						
Cellosyl	34.1	25.7	26.0	4.5	10.9	29.7
LytB	33.5	29.1	29.4	22.5	22.6	38.7
branched monomers (42–60 min)						
Cellosyl	11.4	15.0	7.4	44.9	37.4	16.4
LytB	20.9	23.4	17.2	34.0	26.2	17.3
directly crosslinked dimers (60–69 min)						
Cellosyl	18.2	18.1	23.4	4.3	3.6	6.7
LytB	6.8	8.8	13.0	6.8	9.8	7.7
bridged, branched and bridged-branched dimers (69–88 min)						
Cellosyl	25.6	32.2	28,9	37.2	42.4	31.6
LytB	7.0	11.9	19.5	17.8	16.6	9.2
trimers (88-end min)						
Cellosyl	2.3	4.8	8.9	6.4	2.7	1.1
LytB	0.5	1.0	3.1	1.9	2.1	0.7
