# Supplementary material for: Substrate recognition and catalysis by LytB, a pneumococcal peptidoglycan hydrolase involved in virulence
Source: Sci Rep. 2015 Nov 5;5:16198. doi: 10.1038/srep16198 (PMC4633669; doi:10.1038/srep16198)
Supplement: Supplementary Information [file srep16198-s1.pdf]

Supporting Information

Substrate recognition and catalysis by LytB, a pneumococcal peptidoglycan hydrolase involved in virulence

Palma Rico-Lastres[1,2], Roberto Díez-Martínez[2,3], Manuel Iglesias-Bexiga[1,2], Noemí Bustamante[1,2], Christine Aldridge[4], Dusan Hesek[5], Mijoon Lee[5], Shahriar Mobashery[5], Joe Gray[6], Waldemar Vollmer[4], Pedro García[2,3*], Margarita Menéndez[1,2*]

[1]Instituto de Química-Física Rocasolano, Consejo Superior de Investigaciones Científicas, Serrano 119, 28006, Madrid, Spain.

[2]CIBER de Enfermedades Respiratorias (CIBERES).

[3]Centro de Investigaciones Biológicas, Consejo Superior de Investigaciones Científicas, Ramiro de Maeztu 9, 28040, Madrid, Spain.

[4]Centre for Bacterial Cell Biology, Institute for Cell and Molecular Biosciences, Newcastle University, Newcastle upon Tyne NE2 4AX, United Kingdom.

[5]Deparment of Chemistry and Biochemistry, University Notre Dame, Notre Dame, Indiana 46556, United States.

[6]Institute for Cell and Molecular Biosciences, Newcastle University, Newcastle upon Tyne NE2 4AX, United Kingdom.

Correspondence to: Margarita Menéndez (mmenendez@iqfr.csic.es) or Pedro García (pgarcia@cib.csic.es).

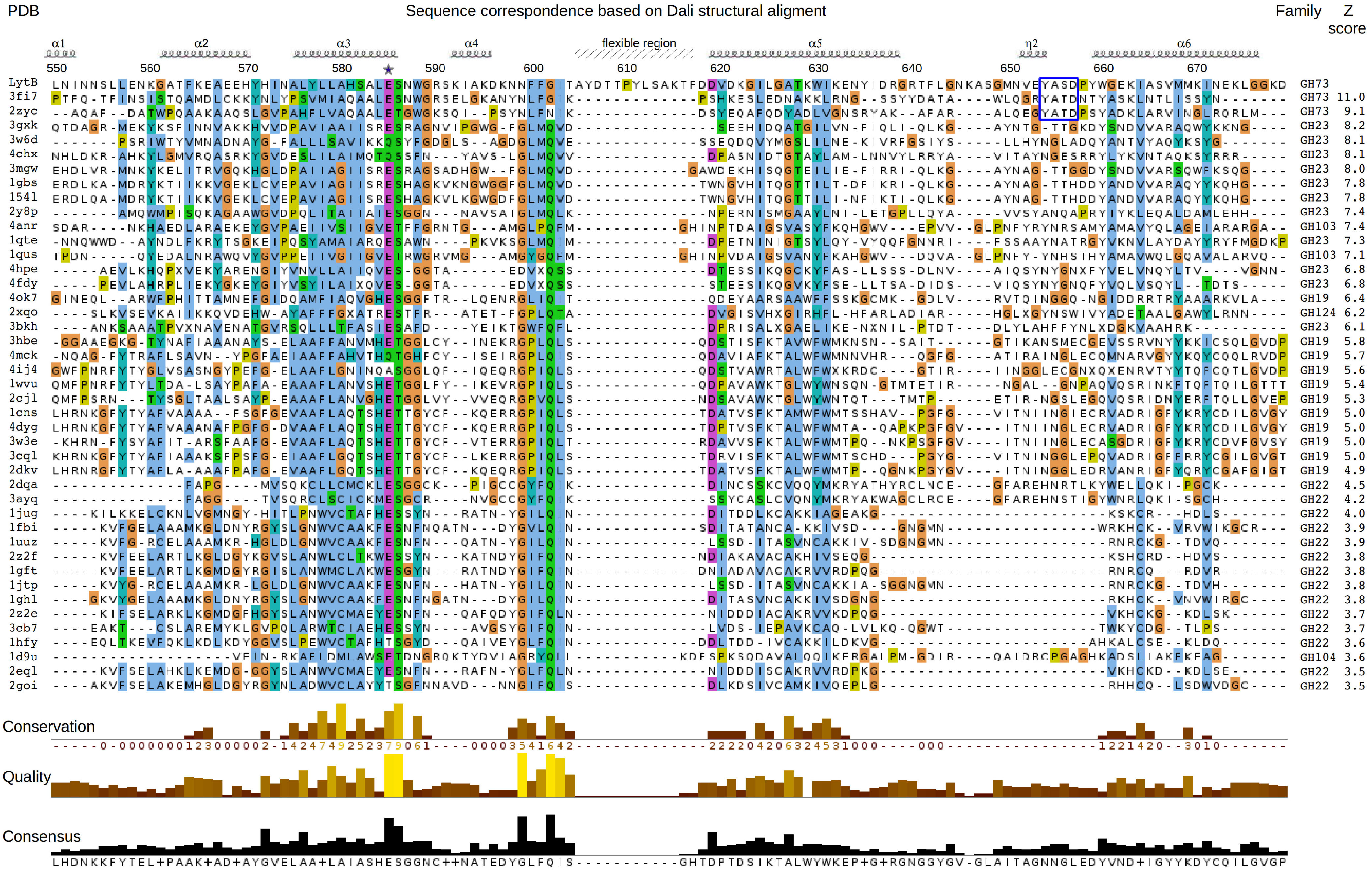

Supplementary Figure S1

Supplementary Figure S1. 3D Structure-based sequence alignment of LytB$_{GH73}$ (PDB 4Q2W) with related proteins identified by the Dali server[1]. Sequences are sorted by structural similarity with LytB (Z-score). GH families are indicated on the right. Secondary structural elements are indicated at the top of the alignment drawn with JalView[2]. Amino acids are coloured by side chain properties. The star indicates the invariant glutamic acid acting as proton donor and the blue box the conserved YA(S/T)D motif of the GH73 family. Bottom histograms show statistics on alignment quality and consensus annotations, and the consensus sequence.

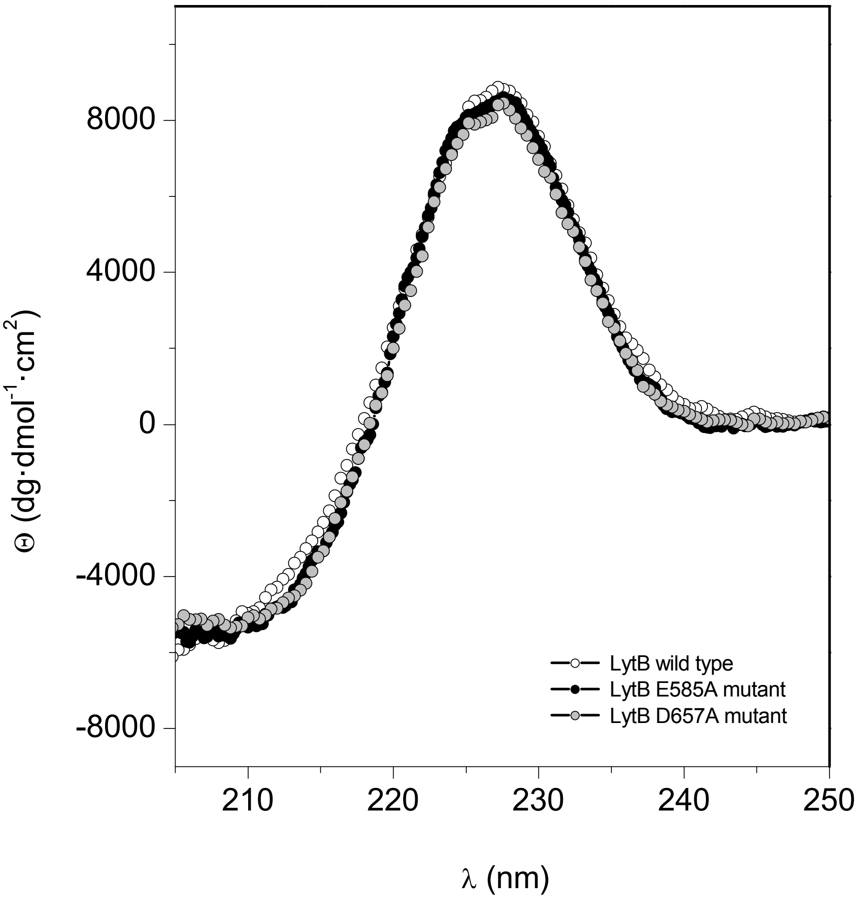

Supplementary Figure S2. Comparison of the far-UV CD spectra of LytB wild type and the E585A and D657 mutants. Measurements performed at 20ºC in Pi buffer (pH 8.0) at 0.16 mg/ml protein. Data were transformed into average molar ellipticities per residue.

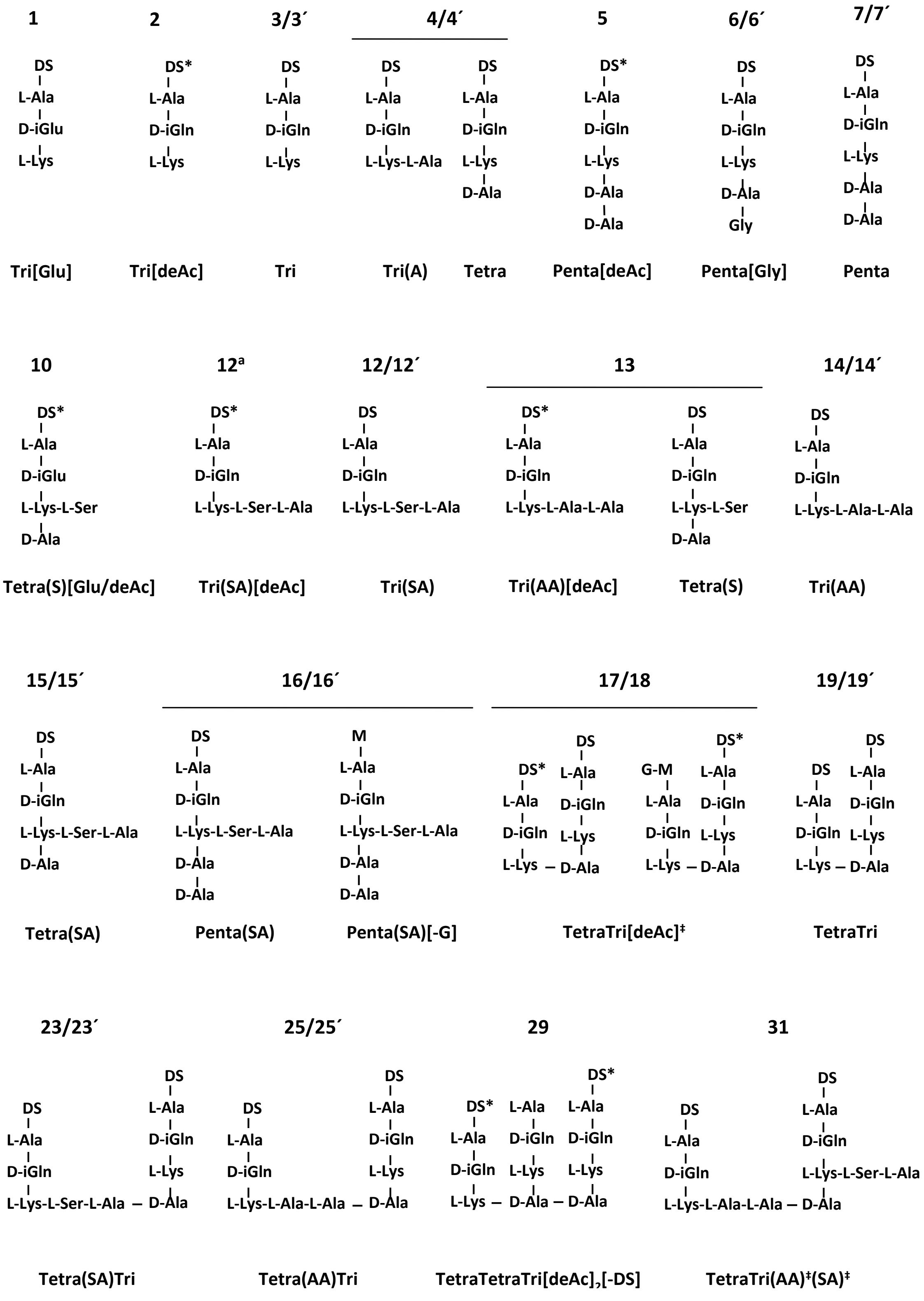

Supplementary Figure S3. Proposed structures of the fragments solubilized by treatment of *S. pneumoniae* cell wall with the glucosaminidase LytB or the PG with the muramidase cellosyl. DS (disaccharide unit) stands for GlcNAc-MurNAc(r) (*N*-acetyglucosamine-*N*-acetylmuramitol) in cellosyl products, and MurNAc-GlcNAc(r) (*N*-acetylmuramic acid-*N*-acetylglucosaminitol) in those of LytB (numbers with apostrophe); DS*, disaccharide unit with a deacetylated glucosamine residue (instead of *N*-acetylglucosamine). Peak numbering as in Bui *et al.*, 2012[3].

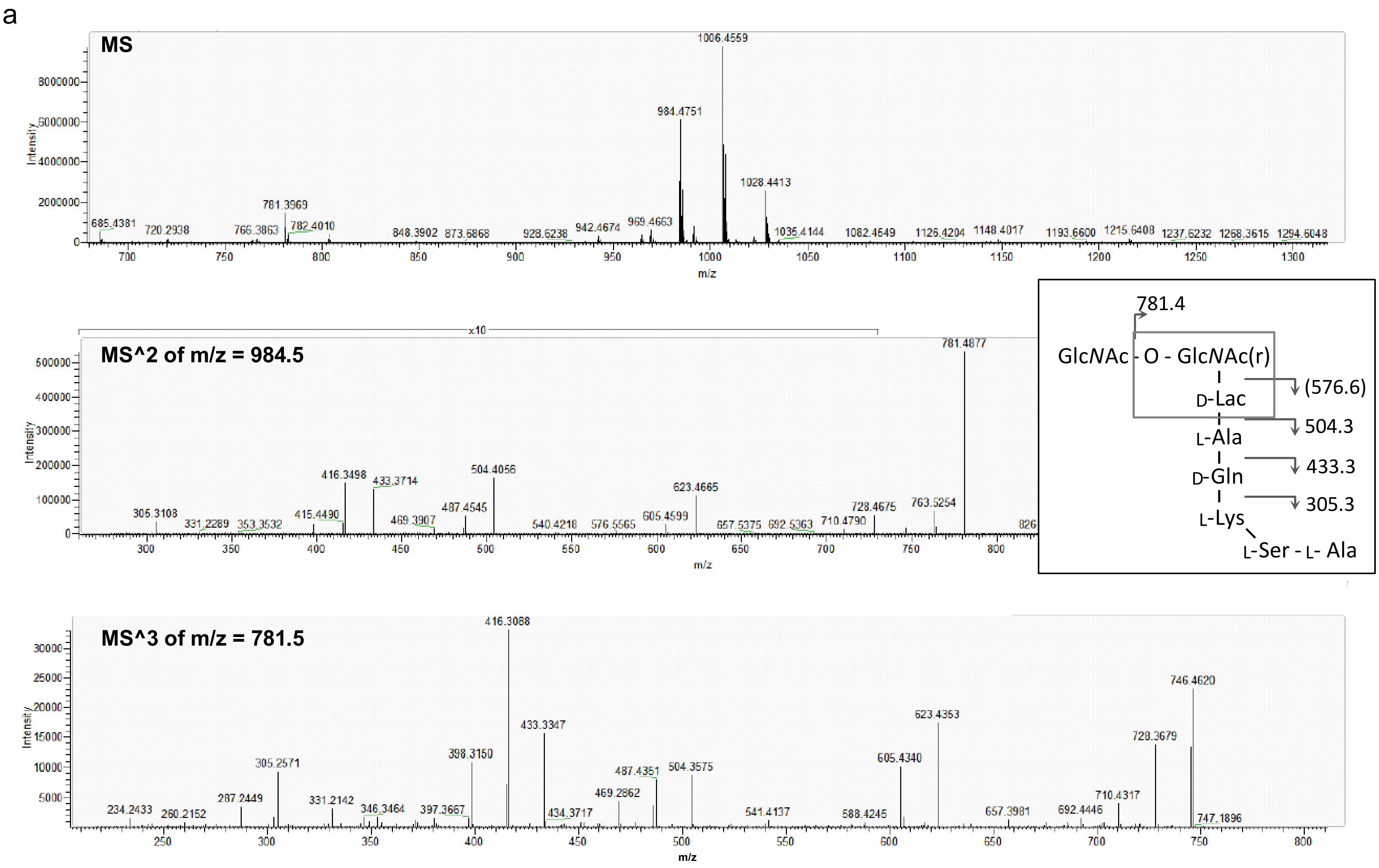

Supplementary Figure S4

b

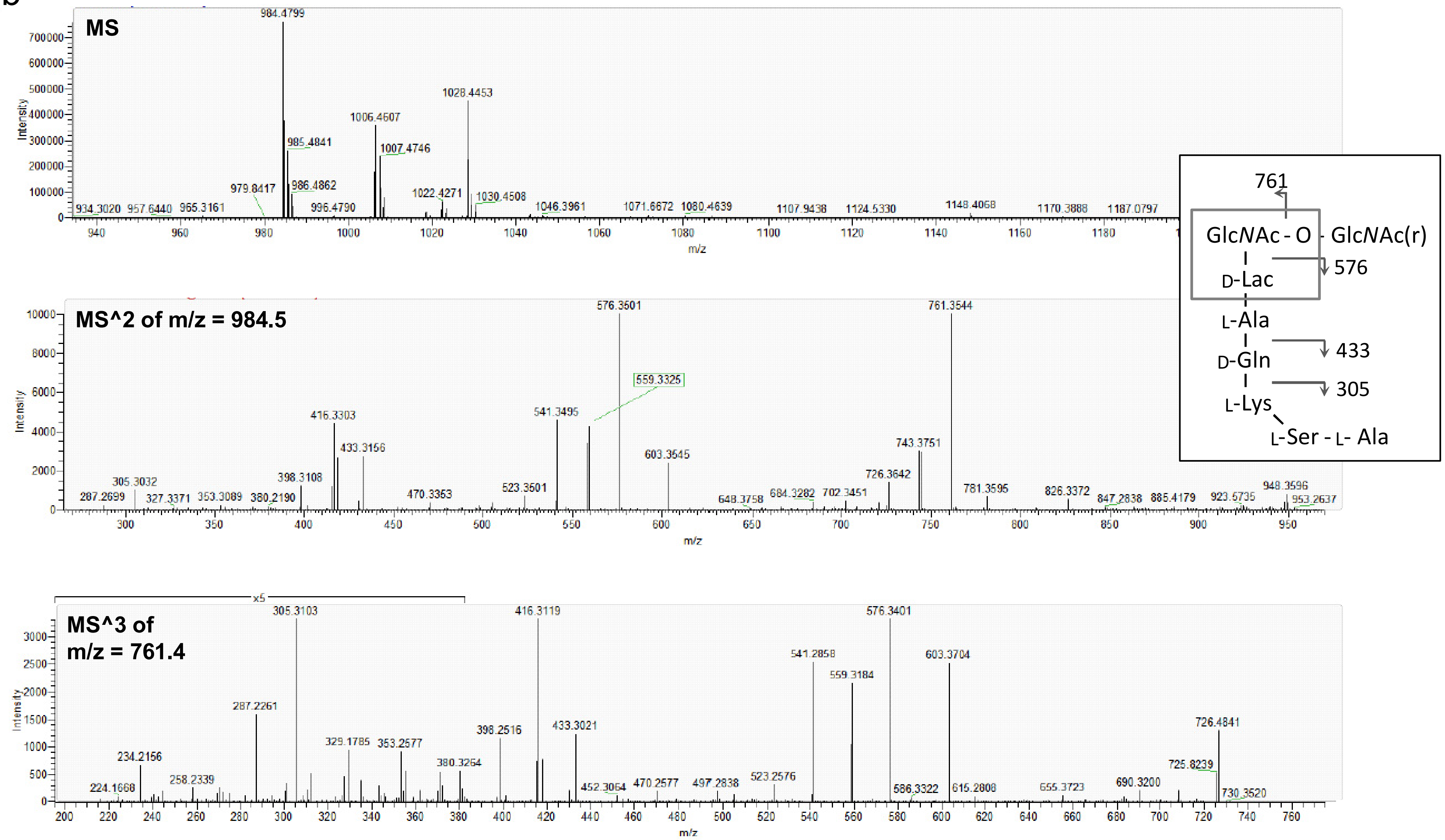

Supplementary Figure S4. MS analysis of isomeric PG fragments obtained by Cellosyl or LytB. (a) MS and MS fragmentation spectra of peak 12 (Tri(SA)) obtained by cellosyl digest followed by reduction with NaBH4. The intense ion with m/z = 781, the weak ion with m/z = 576 and the ion with m/z = 504 are all consistent with the presence of a reduced *N*-acetylmuramitol residue [Mur*N*Ac(r), corresponding to Glc*N*Ac(r)-D-Lac, grey box in the inset]. (b) LytB released a fragment with identical mass but different MS fragmentation pattern (peak 12'). The intense ion with m/z = 761 and an intense lactoyl-peptide ion (m/z = 576), obtained with high intensity only from fragments containing non-reduced Mur*N*Ac (grey box) are consistent with the presence of a reduced Glc*N*Ac(r) residue. This analysis shows that LytB is an *N*-acetylglucosaminidase, and cellosyl is an *N*-acetylmuramidase.

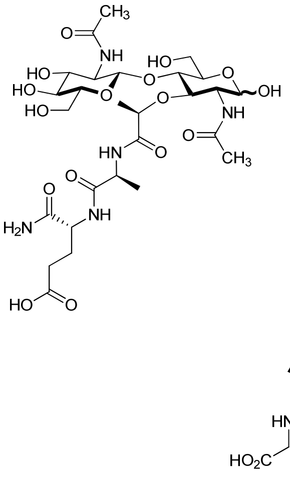

GMDP

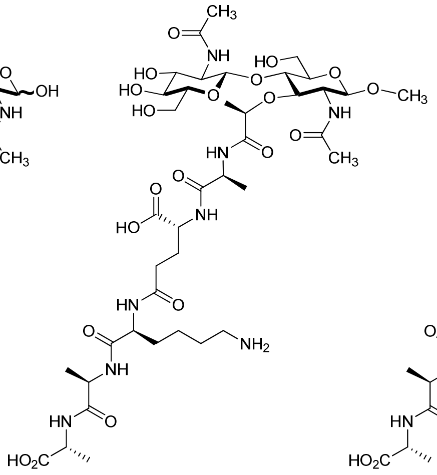

GMPP

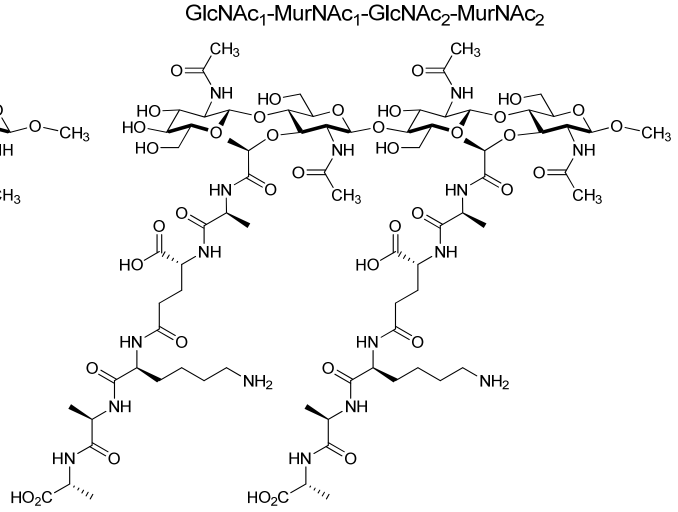

(GMPP)$_2$

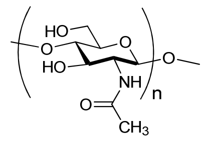

(GlcNAc)$_n$

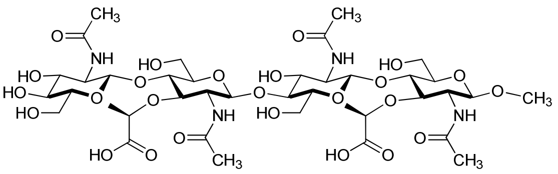

(GM)$_2$

Supplementary Figure S5. Schematic representation of PG analogues used in this work. The numbering of (GMPP)$_2$ sugar units used in docking studies is indicated in the figure labels.

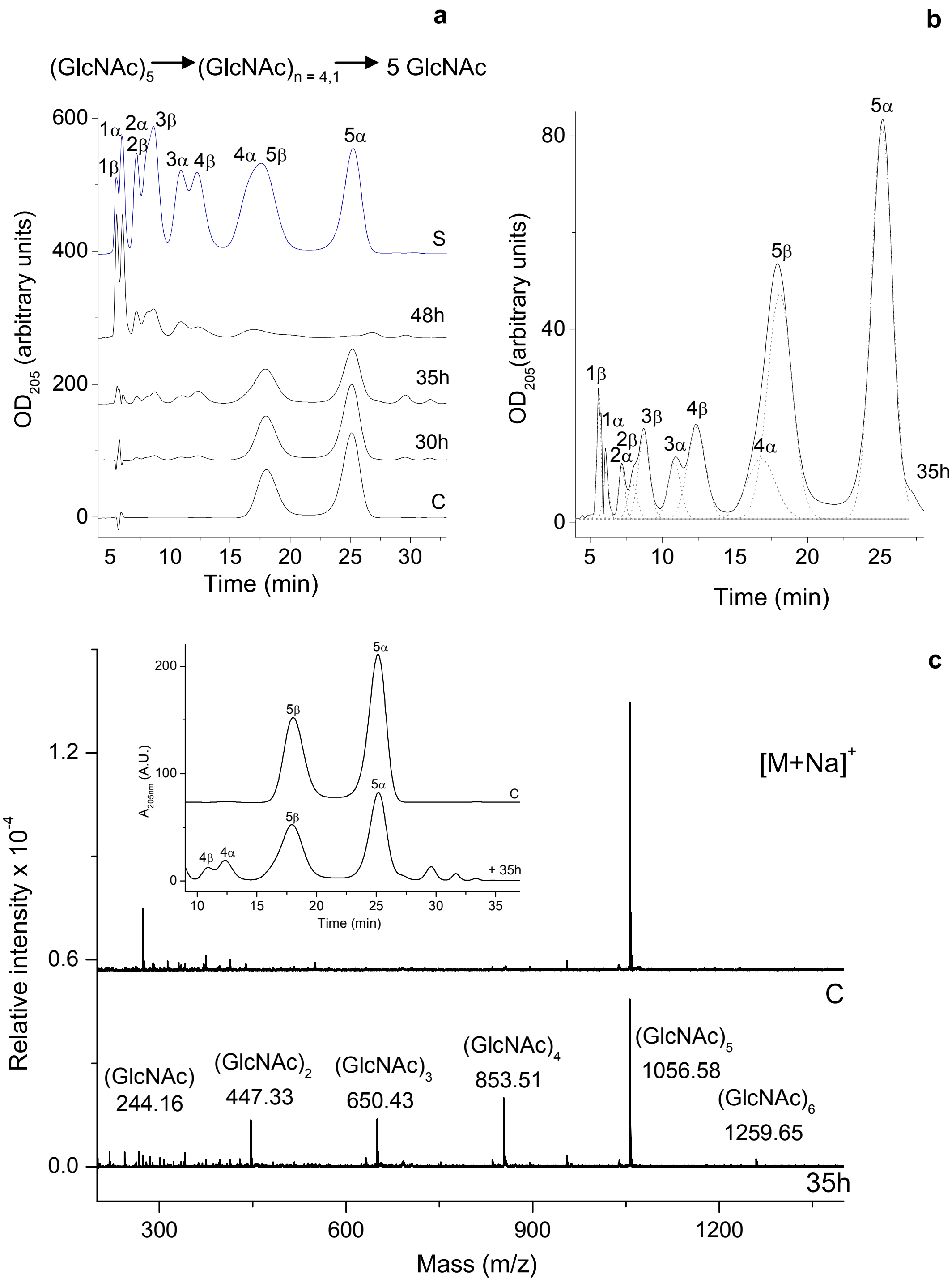

Supplementary Figure S6. HPLC and MALDI-TOF analysis of $(GlcNAc)_5$ products upon treatment with LytB. The reaction was carried out at 37°C in Pi buffer, pH 7.0. Aliquots of the reaction sample were withdrawn at different times along the hydrolysis reaction. (a) Incubation of 1.5 mM $(GlcNAc)_5$ with LytB (8.4 µM). (b) Deconvolution of the elution profile after 35h of incubation with LytB. (c) Detection of glycosyltransferase products by HPLC and MALDI-TOF after 35h of incubation. Product masses (*m/z*) correspond to the $[M+Na]^+$ species of $(GlcNAc)_6$ together with the hydrolysis products. Monoisotopic masses $[M+Na]^+$ for each compound are indicated. C traces correspond to controls without protein at the longest incubation time. Peaks labelled as in Fig. 4 (main text)

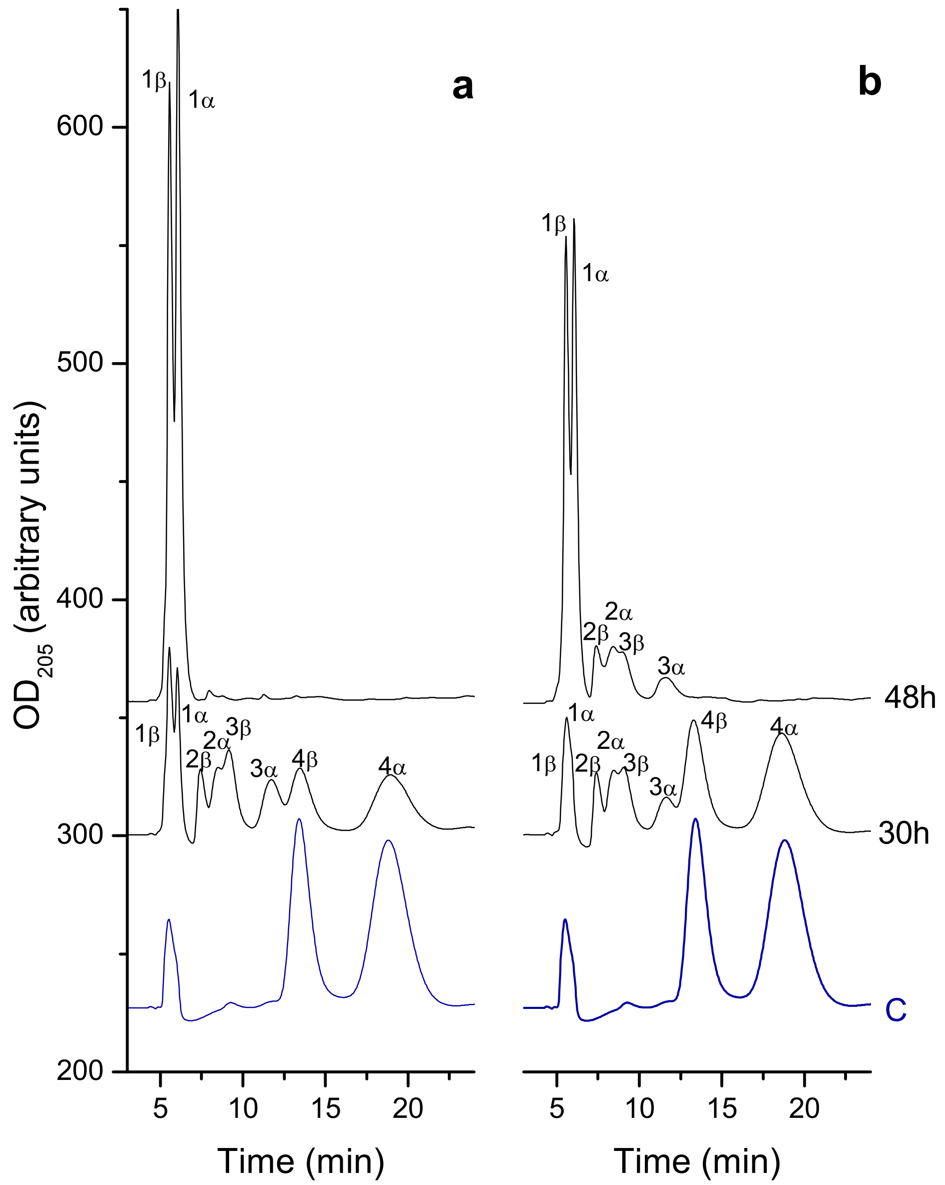

Supplementary Figure S7. HPLC analysis of $(GlcNAc)_4$ products upon treatment with LytB wild type and D657A protein mutant. The reaction was carried out at 37°C in Pi buffer, pH 7.0, during 48 hours. Aliquots of the reaction sample were withdrawn at different times along the hydrolysis reaction. (a) Hydrolysis of $(GlcNAc)_4$ (1.5 mM) by LytB wild type (4.0 μM) and (b) by D657A mutant protein (4.0 μM). C traces correspond to controls without protein at the longest incubation time. Peaks labelled as in Fig. 4 (main text)

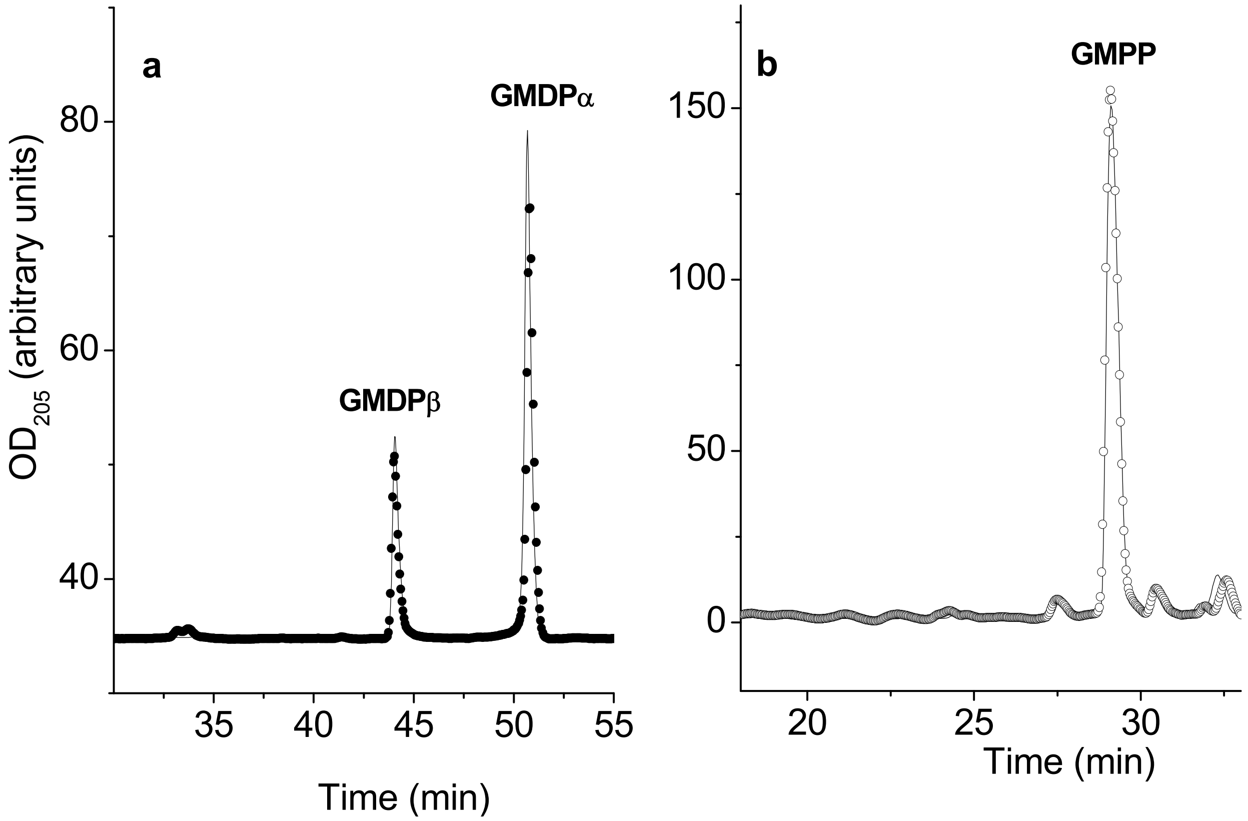

Supplementary Figure S8. HPLC analysis of LytB activity on GMDP and GMPP. The incubation was carried out at 37°C in Pi buffer (pH 7.0) and aliquots of the mixture were withdrawn at different times and analysed by HPLC on a reversed-phase column (*Tracer Excel 120 ODS-B*, 250 × 4 mm, 5µm). Samples were eluted at 20°C using a 40 min gradient from 2% to 15% of acetonitrile in 0.1% trifluoroacetic acid. Continuous traces and symbols correspond to controls and samples after 48 h of incubation, respectively. *α* and *β* refer to GMDP alpha and beta anomers. (a) Incubation of GMDP (106 µM) with LytB (5.3 µM). (b) Incubation of GMPP (200 µM) with LytB (5.6 µM).

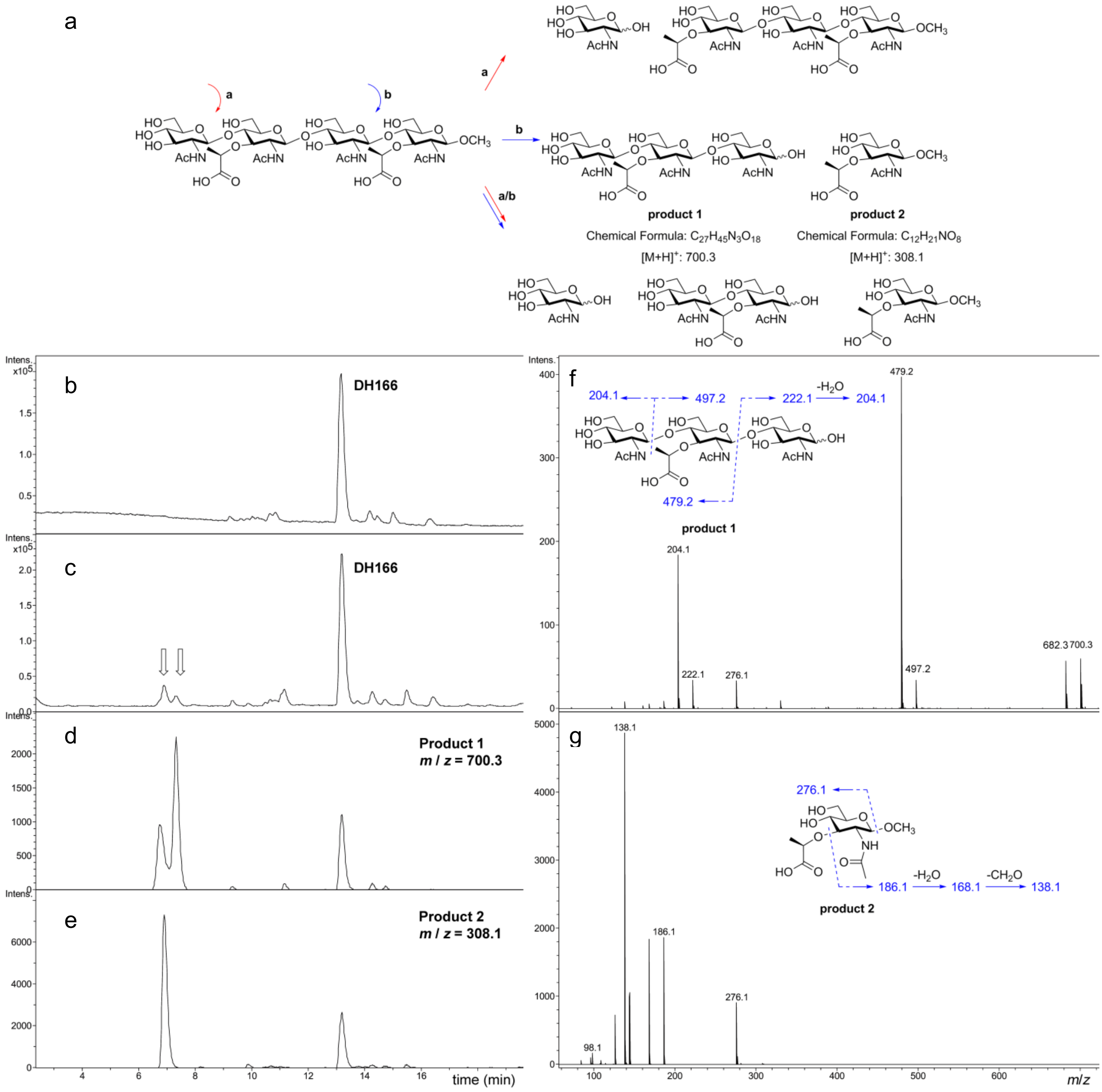

Supplementary Figure S9. LC/MS and LC/MS/MS analysis of LytB activity on (GM)$_2$. The incubation was carried out at 37°C in Pi buffer (pH 7.0) and aliquots of the mixture were withdrawn at different times and analysed by UPLC on a reversed-phase column (Acquity UPLC HSS T3, 150 × 2.1 mm, 1.8 µm, Waters). Samples were eluted at 40°C using a 15 min gradient from 0% to 5% of acetonitrile in 0.1% formic acid. (a) Possible reaction products of LytB on (GM)$_2$. Among three possibilities (I, II or I/II), the reaction of LytB goes by route II. (b) LC/MS total-ion chromatograms of (GM)$_2$ at time = 0 h and (c) at time = 48 h of incubation of (GM)$_2$ (1.5 mM) with LytB (4.0 µM). (d) LC/MS extracted-ion chromatograms of Product 1 at *m*/*z* = 700.3 and (e) Product 2 at *m*/*z* = 308.1. (f) Collision-induced spectra (MS/MS spectra) of Product 1 and (g) Product 2.

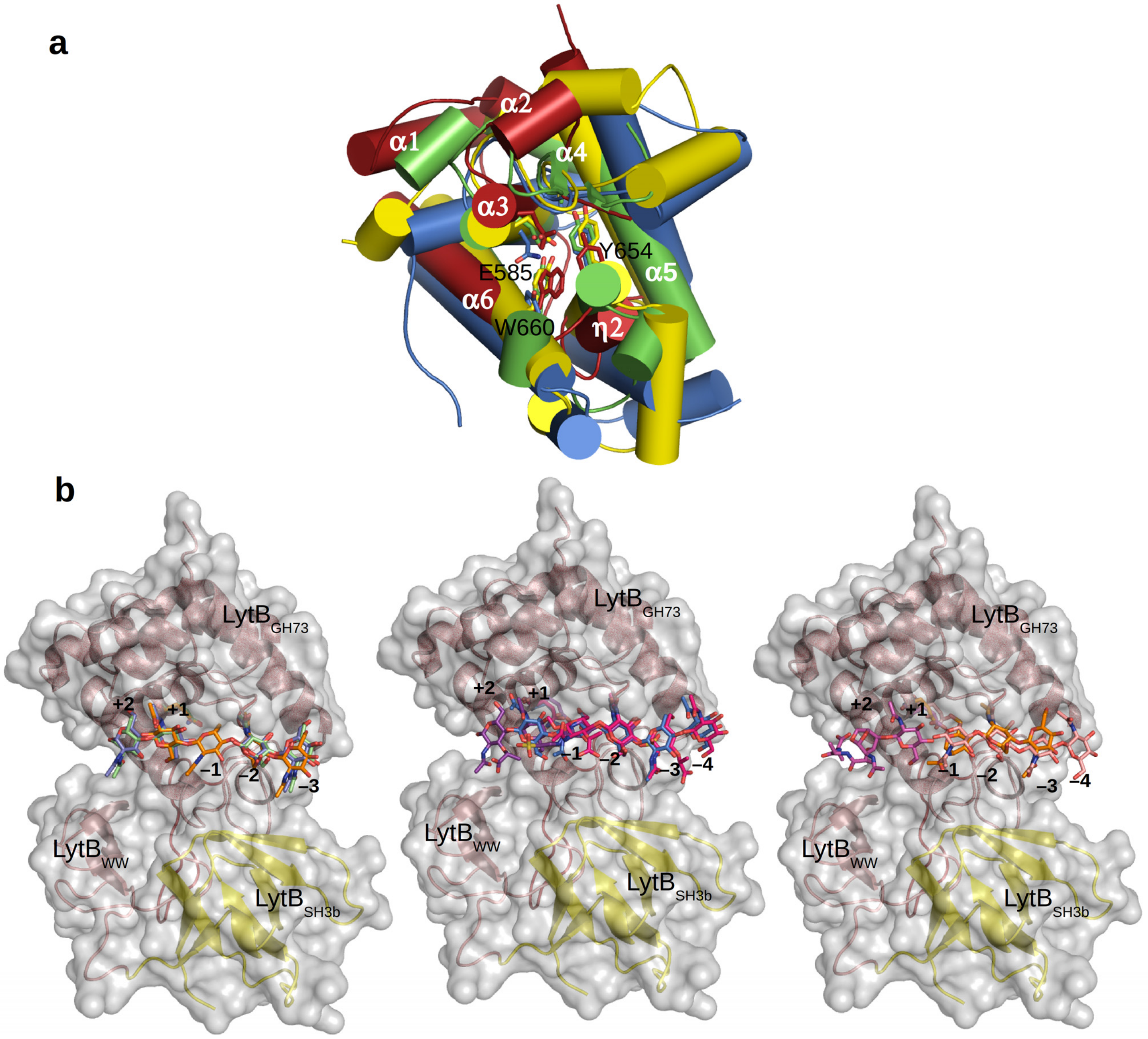

Supplementary Figure S10. Structural comparison of the LytB$_{CAT}$ with related enzymes of the GH23 family. (a) Superimposition of secondary structural elements labelled as in LytB$_{CAT}$ structure. Helices are shown as cylinders. Catalytic proton donor (E585 in LytB) and conserved aromatic residues (W660 and Y664 in LytB) are in stick representation. Colour codes: green, Australian black swan G-type lysozyme (PDB 1LSP); blue, Slt70 from *E. coli* (PDB 1QTE), yellow, MltE from *E. coli* (PDB 4HJV), and red, LytB$_{CAT}$ (PDB 4Q2W). (b) Surface representation of LytB$_{CAT}$ (grey) with different glycan ligands (coloured sticks) docked into the active site by direct superposition with complexes of related GH23 proteins. Left panel, Superimposition with Ra-ChiC chitinase bound to two (GlcNAc)$_2$ units (blue and green; PDBs 3W6C and 3W6F) or (GlcNAc)$_4$ (orange; PDB 3W6D). Central panel, superposition with MltC from *E. coli* bound to (GlcNAc-MurNAc)$_2$ (pink; PDB 4CFP) or (GlcNAc)$_5$ (blue; PDB 4HJC), and MltE in complex with bulgecin and GMDD (purple; PDB 4HJV). Right panel, superposition with Slt70 in complex with a 1,6-anhydromurotripeptide (magenta; PDB 1QTE), phiKZ transglycosylase bound to (GlcNAc)$_4$ (light pink; PDB 3BKV); and the catalytic domain of RpfB from *Mycobacterium tuberculosis* bound to (GlcNAc)$_3$ (orange; PDB 4KPM). Numbering indicates subsites (from –4 to +2) occupied by the saccharide units in the solved complexes.

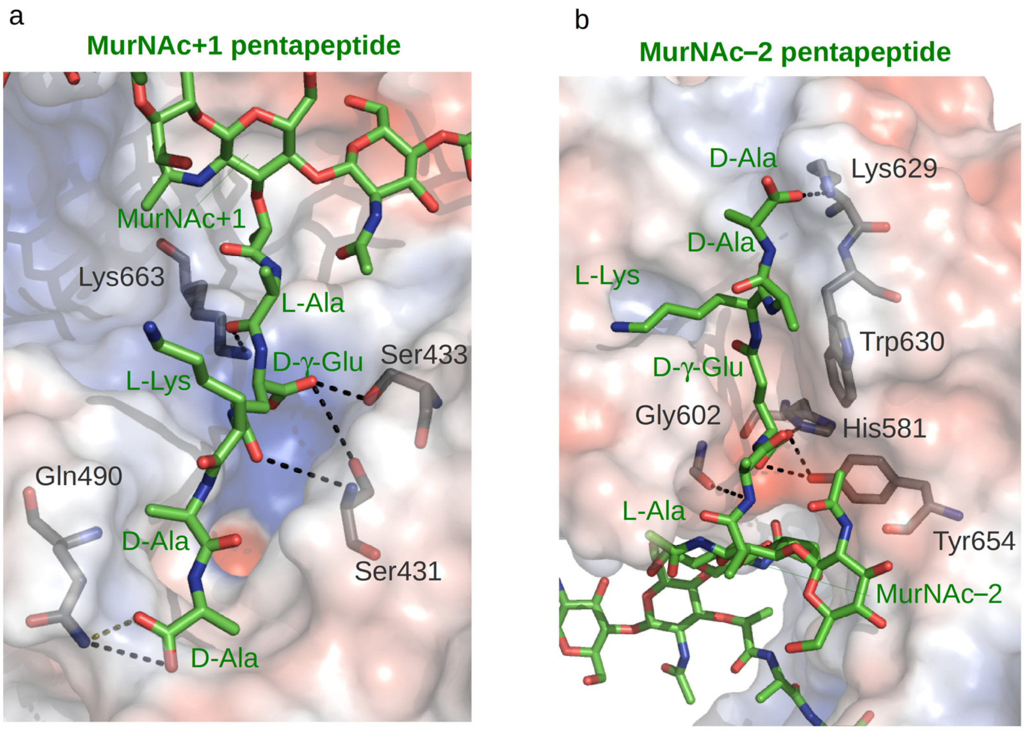

Supplementary Figure S11. Putative interactions mediated by the stem peptides in the $LytB_{CAT}/(GMPP)_3$ complex model. Contact networks of MurNAc+1 pentapeptide (a) and MurNAc–2 pentapeptide (b). LytB residues making contacts are shown as grey sticks and the stem peptides in green. The protein surface is coloured according to the electrostatic potential and the feasible hydrogen bonds are indicated with yellow dashed lines.

Supplementary Table S1. Primers used in this study for construction of alanine mutants and truncated variants of LytB

DNA Oligo	Oligonucleotide sequence[a]
E585A Fw	5’-CCATAGTGCGCTAG**CG**AGTAACTGGGGAAG-3’
E585A Rv	5’-CTTCCCCAGTTACT**CG**CTAGCGCACTATGG-3’
D596A Fw	5’-GGGGAAGAAGTAAAATTGCCAAAG**CG**AAGAATAATTTCTTTGG-3’
D596A Rv	5’-CCAAAGAAATTATTCTT**CG**CTTTGGCAATTTTACTTCTTCCCC-3’
D607A Fw	5’-TTTGGCATTACAGCCTATG**CG**ACGACCCCTTACC-3’
D607A Rv	5’-GGTAAGGGGTCGT**CG**CATAGGCTGTAATGCCAAA-3’
D618A Fw	5’-CTGCCAAGACATTTG**CG**GACGTGGATAAGGG-3’
D618A Rv	5’-CCCTTATCCACGTC**C**GCAAATGTCTTGGCAG-3’
D619A Fw	5’-CCTTTCTGCCAAGACATTTGATG**CG**GTGGATAAGGGAATTTTAGG
D619A Rv	5’-CCTAAAATTCCCTTATCCAC**CG**CATCAAATGTCTTGGCAGAAAGG-3’
D621A Fw	5’-CCAAGACATTTGATGATGTGG**CG**AAGGGAATTTTAGGTGCAACC-3’
D621A Rv	5’-GGTTGCACCTAAAATTCCCTT**CG**CCACATCATCAAATGTCTTGG-3’
E633A Fw	5’-GGTGCAACCAAGTGGATTAAGG**CG**AATTATATCGATAGGGGAAG-3’
E633A Rv	5’-CTTCCCCTATCGATATAATT**CG**CCTTAATCCACTTGGTTGCACC-3’
D637A Fw	5’-GGATTAAGGAAAATTATATCG**CG**AGGGGAAGAACTTTCCTTGG-3’
D637A Rv	5’-CCAAGGAAAGTTCTTCCCCT**CG**CGATATAATTTTCCTTAATCC-3’
D653A Fw	5’-GGCTTCTGGTATGAATGTGG**CG**TATGCTTCAGACCCTTATTGGG-3’
D653A Rv	5’-CCCAATAAGGGTCTGAAGCATA**CG**CCACATTCATACCAGAAGCC-3’
D657A Fw	5’-GGTATGAATGTGGAATATGCTTCAG**CG**CCTTATTGGGGC-3’
D657A Rv	5’-GCCCCAATAAGG**CG**CTGAAGCATATTCCACATTCATACC-3’
E662A Fw	5’-GCTTCAGACCCTTATTGGGGCG**CG**AAAATTGCTAGTGTGATGATG-3’
E662A Rv	5’-CATCATCACACTAGCAATTTT**CG**CGCCCCAATAAGGGTCTGAAGC-3’
E673A Fw	5’-GTGTGATGATGAAAATCAATG**C**GAAGCTAGGTGGCAAAGATTAG-3’
E673A Rv	5’-CTAATCTTTGCCACCTAGCTTC**G**CATTGATTTTCATCATCACAC-3’
LytBΔ1	5’- CGGGATCCGGGAATGAGTGGGTTTTTGATAC-3’
LytBΔ2	5’- CGGGATCCAAATCAGAATGGGTAGAAGACAAG-3’
LytBΔ3	5’- CGGGATCCCACTATTATTATCTAAAATCCGGTG-3’
LytBΔ4	5’- CGGGATCCGCCAATGAATGGATTTG-3’
LytBΔ5	5’- CGGGATCCAATGAGACAGTAGATGGTTATCAG-3’
LytBΔ1NdeI	5’- GTAAATATCATATGGCGAATGAGTGGGTTTTTGATAC-3’
LytBΔ2NdeI	5’- GTAAATATCATATGAAATCAGAATGGGTAGAAGACAAG-3’
LytBΔ3NdeI	5’- GTAAATATCATATGCACTATTATTATCTAAAATCCGGT-3’
LytBΔ4NdeI	5’- GTGATTATCATATGGCCAATGAATGGATTTG-3’
LytBΔ5NdeI	5’- GTAAATATCATATGAATGAGACAGTAGATGGTTATCAG-3’
LytB52	5’- AACTGCAGTTAATCTTTGCCACCTAGCTTC-3’

[a] Bold and underlining indicate mutated nucleotides or the restriction sites introduced for cloning purposes, respectively.

Supplementary Table S2. MS analysis of HPLC fractions from cell wall or PG degradation with LytB or cellosyl

Peak No.[1]	Proposed structure[2]	Theoretical neutral mass (Da)	Measured neutral mass (Da)
2	Tri[deAc]	783.386	783.44
3	Tri	825.397	825.45
3’	Tri	825.397	825.41
7’	Penta	967.471	967.50
10	Tetra(S)[Glu/deAc]	942.435	942.47
12[a]	Tri(SA) [deAc]	941.455	941.46
12	Tri(SA)	983.466	983.48
12’	Tri(SA)	983.466	983.49
13	Tri(AA)[deAc]	925.460	925.36
14	Tri(AA)	967.471	967.48
14’	Tri(AA)	967.471	967.59
15	Tetra(SA)	1054.503	1054.51
15’	Tetra(SA)	1054.503	1054.60
16’	Penta(SA)	1125.540	1125.60
19	TetraTri	1703.820	1703.96
19’	TetraTri	1703.820	1703.71
23	Tetra(SA)Tri	1861.889	1861.85
23’	Tetra(SA)Tri	1861.889	1861.70
29	TetraTri$(SA)_2$	2019.958	2019.94
31	TetraTri(AA)[‡](SA)[‡]	2003.963	2003.97

[1]The numbers correspond to the peaks in the chromatograms shown in Fig. 3 (main text).

[2]Modificacions: [Glu], presence of isoglutamic acid instead of isoglutamine; [deAc], deacetylation of GlcNAc; [‡], position of modifications not known.

Table S3. Polar interactions of $LytB_{CAT}$ discerned from the model of $LytB_{CAT}/(GMPP)_3$

Subsite	Residue	Atom	Residue	Atom	Localization[1]
+3	MurNAc	O6	Asn587	Nδ2	$LytB_{GH73}$ (α-3)
+2	GlcNAc	O2N	Ser591	Oγ	$LytB_{GH73}$ (α-4)
	GlcNAc	O3	Ser591	Oγ	$LytB_{GH73}$ (α-3)
+1	MurNAc	O2N	Asn587	Nδ2	$LytB_{GH73}$ (α-3)
	L-Ala	NH	Asp657	Oδ2	$LytB_{GH73}$ (loop α-3/ η-2)
	L-Ala	CO	Lys663	Nζ	$LytB_{GH73}$ (α-3)
	D-γ-Glu	Oδ2	Ser433	Oγ	$LytB_{SH3b}$ (β-4)
	D-γ-Glu	Oδ2	Ser431	Oγ	$LytB_{SH3b}$ (β-3)
	D-Ala2	O	Gln490	Nδ2	$LytB_{WW}$ (loop β-2/β-3)
	D-Ala2	OXT	Gln490	Nδ2	$LytB_{WW}$ (loop β-2/β-3)
-1	GlcNAc	O3	Ser656	Oγ	$LytB_{GH73}$ (loop α-3/η-2)
	GlcNAc	O5	Glu585	Oε2	$LytB_{GH73}$ (α-6)
	GlcNAc	O6	Glu585	Oε2	$LytB_{GH73}$ (α-6)
	GlcNAc	O6	Phe601	CO	$LytB_{GH73}$ (loop α-4/flexible region)
-2	MurNAc	N2	Gly602	CO	$LytB_{GH73}$ (loop α-4/ flexible region)
	MurNAc	N2	Tyr429	OH	$LytB_{SH3b}$ (β-4)
	L-Ala	NH	Gly602	CO	$LytB_{GH73}$ (loop α-4/ flexible region)
	D-γ-Glu	Oδ1	His581	Nε2	$LytB_{GH73}$ (α-3)
	D-γ-Glu	Oδ1	Tyr654	OH	$LytB_{GH73}$ (3_{10}- η2)
	D-γ-Glu	Oδ2	Tyr654	OH	$LytB_{GH73}$ (α-3)
	D-Ala2	OXT	Lys629	Nζ	$LytB_{GH73}$ (α-5)
-3	GlcNAc	N2	Glu653	CO	$LytB_{GH73}$ (3_{10}- η2)
	GlcNAc	O4	Lys427	Nζ	$LytB_{SH3b}$ (loop β-3/β-4)

[1]LytB domain (secondary structure element)

Supplementary methods

The Dali server[1] was used for 3D structural comparison and Jalview2[2] to represent structure-based sequence alignments. Circular dichroism spectra (average of 4 consecutive scans) were recorded at 20 °C using a JASCO-J810 spectropolarimeter (Jasco Corp.) equipped with a Peltier cell holder. Measurements were performed at a protein concentration of 0.16 mg/ml as reported elsewhere[4].

Supplementary References

1 Holm, L. & Rosenstrom, P. Dali server: conservation mapping in 3D. *Nucleic Acids Res.* **38**, W545-549 (2010).

2 Waterhouse, A. M., Procter, J. B., Martin, D. M., Clamp, M. & Barton, G. J. Jalview Version 2. A multiple sequence alignment editor and analysis workbench. *Bioinformatics* **25**, 1189-1191 (2009).

3 Bui, N. K. *et al.* Isolation and analysis of cell wall components from *Streptococcus pneumoniae*. *Anal. Biochem.* **421**, 657-666 (2012).

4 Bustamante, N. *et al.* Cpl-7, a lysozyme encoded by a pneumococcal bacteriophage with a novel cell wall-binding motif. *J. Biol. Chem.* **285**, 33184-33196 (2010).
